# Wheat rust epidemics damage Ethiopian wheat production: A decade of field disease surveillance reveals national-scale trends in past outbreaks

**DOI:** 10.1371/journal.pone.0245697

**Published:** 2021-02-03

**Authors:** M. Meyer, N. Bacha, T. Tesfaye, Y. Alemayehu, E. Abera, B. Hundie, G. Woldeab, B. Girma, A. Gemechu, T. Negash, T. Mideksa, J. Smith, M. Jaleta, D. Hodson, C. A. Gilligan

**Affiliations:** 1 Visual Data Analysis, Center For Earth System Research and Sustainability, Regional Computing Center, University of Hamburg, Hamburg, Germany; 2 Epidemiology and Modelling Group, Department of Plant Sciences, University of Cambridge, Cambridge, United Kingdom; 3 Ethiopian Institute of Agricultural Research (EIAR), Addis Ababa, Ethiopia; 4 International Maize and Wheat Improvement Center (CIMMYT), Addis Ababa, Ethiopia; 5 Dept. of Plant Pathology, University of Minnesota, St Paul, Minnesota, United States of America; 6 Oromia Agricultural Research Institute, Sinana, Ethiopia; 7 International Maize and Wheat Improvement Center (CIMMYT), Texcoco, Mexico; Institute of Genetics and Developmental Biology Chinese Academy of Sciences, CHINA

## Abstract

Wheat rusts are the key biological constraint to wheat production in Ethiopia—one of Africa’s largest wheat producing countries. The fungal diseases cause economic losses and threaten livelihoods of smallholder farmers. While it is known that wheat rust epidemics have occurred in Ethiopia, to date no systematic long-term analysis of past outbreaks has been available. We present results from one of the most comprehensive surveillance campaigns of wheat rusts in Africa. More than 13,000 fields have been surveyed during the last 13 years. Using a combination of spatial data-analysis and visualization, statistical tools, and empirical modelling, we identify trends in the distribution of wheat stem rust (Sr), stripe rust (Yr) and leaf rust (Lr). Results show very high infection levels (mean incidence for Yr: 44%; Sr: 34%; Lr: 18%). These recurrent rust outbreaks lead to substantial economic losses, which we estimate to be of the order of 10s of millions of US-D annually. On the widely adopted wheat variety, *Digalu*, there is a marked increase in disease prevalence following the incursion of new rust races into Ethiopia, which indicates a pronounced boom-and-bust cycle of major gene resistance. Using spatial analyses, we identify hotspots of disease risk for all three rusts, show a linear correlation between altitude and disease prevalence, and find a pronounced north-south trend in stem rust prevalence. Temporal analyses show a sigmoidal increase in disease levels during the wheat season and strong inter-annual variations. While a simple logistic curve performs satisfactorily in predicting stem rust in some years, it cannot account for the complex outbreak patterns in other years and fails to predict the occurrence of stripe and leaf rust. The empirical insights into wheat rust epidemiology in Ethiopia presented here provide a basis for improving future surveillance and to inform the development of mechanistic models to predict disease spread.

## Introduction

Ethiopia is the largest wheat-producing country in sub-Saharan Africa [1]. It is estimated that more than 4–5 million smallholder households depend on wheat production, both as a food crop or as a source of income [2]. In most of Ethiopia, rainfed wheat is grown in the highlands by smallholder farmers with an average field size of about 1 hectare. The timing of the wheat growing season varies amongst wheat producing regions. In most parts of the country, the main wheat season follows the long rainy season (*meher*), which starts around June and lasts until December. In some areas, wheat is also grown in the preceding short rainy (*belg*) season, from March or April to June or July. In the main wheat producing areas of Ethiopia (south-eastern and central-western parts of the Ethiopian highlands), the environment is suitable for infection with wheat rusts almost year around [3].

Wheat stem rust and wheat stripe rust are the major biotic constraints to wheat production in Ethiopia. Recurrent epidemics have occurred during the last decades, including a major wheat stripe rust epidemic in 2010 [4] and a large wheat stem rust outbreak caused by race TKTTF in 2013 with yield losses up to 100% and average losses of approximately 50% [5]. Households have developed different coping strategies as a reaction to recurrent wheat rust outbreaks that include increasing the diversity of wheat varieties grown and increasing the wheat area with disease resistant varieties [6]. Disease-resistant wheat varieties are one of the most important means of disease management. During the past six decades > 130 bread or durum wheat varieties have been released in Ethiopia with the aim of increasing yields and improving disease resistance [7]. DNA fingerprinting has shown that recently released rust resistant bread wheat varieties have been widely adopted across Ethiopia [8]. Despite large varietal choice, a limited number (< 20) of varieties cover most of the planted wheat area in any given year.

To advance our understanding of wheat rust epidemiology in Ethiopia, a long-term surveillance campaign was launched in 2007 [9]. Since then, approximately 13,500 field disease surveys have been conducted in a joint effort of the Ethiopian Institute for Agricultural Research (EIAR), Regional Agricultural Research Centers, agricultural universities and the International Maize and Wheat Improvement Centre (CIMMYT). Today, the on-going field disease surveillance efforts are the essential backbone of a near real-time wheat rust early warning system in Ethiopia. The early warning system integrates field surveys, phone surveys, meteorological and epidemiological models to assess risks and to predict wheat rust outbreaks [10]. Recently, disease monitoring was further improved by carrying out in-field gene-based disease diagnostics using MARPLE—a Mobile And Real-time PLant disEase system [11]. Despite the progress made in monitoring and predicting wheat rusts, the diseases continue to be a major biotic constraint to wheat production, not only in Ethiopia but worldwide. Examples of advanced surveillance, monitoring and early warning systems in different parts of the world include: (i) recent efforts to extend the wheat rust early warning system from Ethiopia to countries in South-Asia (ongoing work; unpublished); (ii) the Global Wheat Rust Monitoring System [9,12]; (iii) the ongoing EU Project RustWatch [13].

The study described here was conducted as part of ongoing efforts for controlling wheat rust outbreaks in Ethiopia. It was designed to identify and compare characteristic outbreak patterns for three wheat diseases (stem, stripe and leaf rust) within and between years in Ethiopia using long-term surveillance data. An improved understanding of past rust outbreak patterns and the identification of high-risk areas for disease outbreaks will inform strategies for future surveillance and control of wheat rusts in Ethiopia, by increasing the chances for early disease detection, and by targeting the deployment of fungicides and rust resistant varieties to critical areas for disease control. The results summarized in this paper also serve as an empirical reference for refining and testing the mechanistic models for predicting wheat rusts, as currently integrated into the wheat rust early warning system in Ethiopia [10].

Our analysis is motivated by the following questions: how widely distributed are wheat rusts in Ethiopia? Are there any clear spatiotemporal trends in past disease outbreaks–for example, are there any geographical areas or wheat varieties with particularly high disease levels? How does disease prevalence change over time and are there recurrent epidemic waves starting from a particular location in Ethiopia? What are the likely national-scale economic losses from wheat rusts in Ethiopia based upon the field disease survey dataset? To what extent can simple empirical models be used for predicting wheat rusts on national scales in Ethiopia?

## Materials and methods

### Summary of the wheat rust field surveillance system in Ethiopia

The *RustTracker* field disease surveillance system in Ethiopia was started in 2007 [9]. The surveillance efforts consist of regular field surveys conducted in all major wheat producing regions of Ethiopia using standardised methods [14]. Two measures of disease intensity were recorded in field disease surveys: *incidence* and *severity*. Disease *incidence* represents an estimate of the proportion of wheat plants in a surveyed field that are infected with a particular wheat rust. Disease *severity* is an estimate of the average extent of infection on individual plants. It is estimated using the modified Cobb scale [15] taking an average amongst all sampled plants within a surveyed field. Four classes are recorded for both *severity* and *incidence*: (i) no infection; (ii) low incidence (severity) [1–20%]; (iii) moderate incidence (severity) [20–40%]; (iv) high incidence (severity) [>40%]. In the analyses, we considered both disease measures, *incidence* and *severity*, for all three types of wheat rusts with the aim of capturing key aspects of wheat rust epidemiology in Ethiopia.

The surveys were traditionally conducted towards the end of the main wheat season (September–December), but, in recent years, surveys are also conducted at the beginning of the main wheat season (July-August) and during the minor wheat season (June-July). The specific locations and timings of surveys varied amongst years. There was no recurring surveillance on a set of fixed, representative locations. Instead, varying survey locations were chosen based, in part, on the priority for early detection of outbreaks. Since 2007, some surveys were conducted in almost all wheat producing areas of Ethiopia, but the main surveillance efforts were focussed on key wheat producing areas, notably, the Arsi, Bale, and Shewa—zones. During the first years (2007–2017) of surveillance efforts, teams of surveyors visited different wheat producing areas at different times of the year and reported disease levels on standardized, hand-written surveillance forms. These surveillance reports were then collected, checked, and entered into a digital survey database after the end of the wheat season. Following 2017 disease surveys were increasingly conducted using a surveillance app developed using ODK (https://opendatakit.org/) for digital collection of surveillance results in near-real time. Selected rust samples collected during the surveillance campaigns were analysed by dedicated laboratories to determine races present using standard methodology and nomenclature [16,17]. Details of the race analyses are not part of the study described here, but the influence of key races are highlighted.

### Data cleansing and consistency checks

There are approximately 13,500 survey entries from years 2007–2019 in the *RustTracker* dataset. For each entry, a set of data attributes is available describing the wheat field that was surveyed. Data attributes for each surveyed wheat field include: date of the survey, location (latitude, longitude, altitude), and size of the wheat field, growth stage of the wheat crop, wheat variety, and wheat rust disease incidence and severity (reported separately for stem rust, stripe rust and leaf rust).

The following data cleansing measures were conducted to improve data quality and consistency. We removed observations that lacked entries in key data attributes (date, location, disease scores). Incorrect and inconsistent data entries were also removed. These included: survey coordinates located outside of Ethiopia or in a lake; disease scores outside the permitted range; survey dates outside the permitted time-interval 2007–2019. Duplicate entries were also removed. We conducted the data cleansing separately for each wheat rust.

The dataset for analysis was further reduced by restriction to years 2010–2019, for which we have >1000 observations per year. Prior to that, there had been problems in standardisation during 2007–2008 as the surveillance programme was introduced, and only around 500 raw observations were available per year. The data in 2009 were confounded by conflating records for no disease and no record, which together accounted for more than 50% of samples. Hence, to ensure good quality of the survey data, we exclude the data prior to 2010, leaving approximately 10,500 clean survey entries covering the ten years from 2010–2019, which provided 10,300, 10,400, and ~10,100 entries, respectively, for stem, stripe and leaf rust.

### Geographical data analysis and visualization

For the comparative analysis of national-scale outbreak patterns of wheat stem, stripe, and leaf rust, we used a combination of spatial data analysis and visualization, statistical tools, and empirical modelling. As a first step, wheat rust incidence and severity scores were mapped separately for each wheat rust using R as a GIS [18], with particular use of packages *raster* [19], *rgdal [20]*, *rcolorbrewer [21]* and *tmap [22]*. The maps with temporal snapshots of disease were combined to obtain time-lapse animations summarizing ten years of wheat rust disease surveillance (S1–S12 Movies). The methods for detailed analysis of past outbreak patterns include: calculation of the *Morans-I* statistic for testing spatial autocorrelation and a ‘hotspot’ analysis based on the Getis-Ord Gi* statistic (calculated using the R package *spdep [23])*; Chi-Square tests, regression analyses and a Receiver Operating Characteristic (ROC) analysis for testing the performance of simple empirical models for predicting wheat rusts in Ethiopia (using the statistics and machine learning toolbox in MATLAB [24]). Details of the spatial analysis of wheat rust outbreaks are given in S1 Appendix.

### Estimation of financial losses caused by wheat rusts

Using the field disease survey dataset in combination with national-scale FAO statistics [1] about wheat production and wheat prices in Ethiopia, we estimate financial income losses, *L_rt_*, due to wheat rust type, *r*, in year, *t*, as follows:

$$L_{rt}=A_{rt}(i)\times Y_{rt}(s,g)\times P_{t}.$$ (1)

The approximate total area infected with wheat rust, *r*, in year, *t*, denoted as, *A_rt_* [ha], is obtained as a function of the annual sample mean disease incidence (considering only moderate and high infections), *i*, and the annual FAO statistic for total national wheat areas in Ethiopia [1]. The approximate yield that is lost due to wheat rust, *r*, in year, *t*, denoted as, *Y_rt_*, is estimated, based on the average wheat yield [tonnes / ha] in Ethiopia, as reported in FAO statistics, and the fraction of yield lost to a specific rust type, *r*. The fraction of yield lost to rust, *r*, is obtained as a function of field-scale disease severity, *s*, and wheat growth stage, *g*, combined with published empirical results [25]. The wheat price, *P_t_*, per tonne of wheat in year, *t*, is taken from yearly FAO statistics [1]. For our estimates of losses caused by wheat rusts we consider only data entries with moderate or high disease incidence and severity scores, because losses on fields with low severity / incidence are usually negligible. Details of the estimation methodology are described in S2 Appendix.

In addition to the total losses caused by wheat rusts, *L_rt_*, (see Eq 1; in units of millions US-D), we also estimate losses relative to the total financial value of all wheat produced per year at market price. The relative losses, *R_rt_*, caused by wheat rust type, *r*, in year, *t*, are obtained as the proportion of financial losses due to rusts relative to the total financial value of all wheat grown in Ethiopia (*L_rt_/M_t_*) The total financial value of wheat produce at market price per year, *M_t_*, is estimated directly from FAO statistics as $M_{t}={\bar{A}}_{t}\times {\bar{Y}}_{t}\times P_{t}$, with ${\bar{A}}_{t}$ denoting the total area of wheat harvested per year; ${\bar{Y}}_{t}$ denoting the annual average yield and, *P_t_*, denoting the wheat price in year, *t* (all reported in [1]).

The methods described above for data cleansing, geographical and statistical data analyses, visualization, and estimation of loss estimates, were implemented in a set of handwritten R (version 3.6.2) and MATLAB (version 2019b) scripts and functions. All scripts along with the survey dataset used for the current study are available on Gitlab [26].

## Results

S1–S6 Movies show time-lapse animations of maps with yearly snapshots of disease severity and incidence for each of the three rusts (S1–S6 Movies). S7–S12 Movies show time-lapse animations of maps with bi-weekly snapshots of disease severity and incidence for each wheat rust to illustrate within-season wheat rust disease progress in all years 2010–2019 (S7–S12 Movies).

The following sections summarize the analysis of the survey data. Our analyses adapt the following sequence: aggregated wheat rust disease levels in all surveys from years 2010–2019; analysis of spatial trends; disease prevalence on different wheat varieties; temporal trends; spatiotemporal trends; estimation of financial losses caused by wheat rusts in Ethiopia; testing the performance of two simple logistic models for predicting wheat rusts on national scales in Ethiopia.

### Aggregated wheat rust disease prevalence in Ethiopia during 2010–2019

*Table 1* summarizes aggregated disease scores for each wheat rust. Analysing the total number of fields in different disease categories in all years 2010–2019 shows highest levels of infection with wheat stripe rust, followed by high levels of wheat stem rust infection and lower levels of wheat leaf rust infection. Two aggregated scores are provided: all surveys with any non-zero disease incidence (severity) were counted (see column “disease present” in *Table 1*) and all surveys with moderate or high incidence (severity) were counted (see column “sum mod/high” in *Table 1*). The aggregated total proportion of positives (with disease scores ≥ low) in all surveys from 2010–2019 is: ~44% for wheat stripe rust, ~34% for wheat stem rust and ~18% for wheat leaf rust.

**Table 1:** Aggregated wheat rust prevalence in Ethiopia during years 2010–2019.

Disease	Total records	Disease absent	Disease present	Incidence				Severity			
				low [<20%]	moderate [20–40%]	high [>40%]	Sum mod/high	low [<20%]	moderate [20–40%]	high [>40%]	Sum mod/high
**Stripe rust**	10 446	5 836 (56%)	4 610 (44%)	1 813 (17%)	593 (6%)	2 204 (21%)	2 797 (27%)	2 709 (26%)	868 (8%)	1 033 (10%)	1 901 (18%)
**Stem rust**	10 343	6 811 (66%)	3 532 (34%)	1 730 (17%)	464 (4%)	1 338 (13%)	1 802 (17%)	2 469 (24%)	571(5%)	492 (5%)	1 063 (10%)
**Leaf rust**	10 149	8 291 (82%)	1 858 (18%)	783 (8%)	342 (3%)	733 (7%)	1 075 (10%)	1307 (13%)	322 (3%)	229 (2%)	551 (5%)

The table shows the number of fields in each disease category (columns) for all three wheat rusts (rows) and both disease measures (incidence and severity). The column “sum mod/high” summarizes the total number of fields with moderate or high disease levels.

### Spatial analysis of wheat rust outbreaks in Ethiopia

For the analysis of spatial trends in past wheat rust outbreaks in Ethiopia the data from all years are aggregated and we focus exclusively on spatial aspects. Temporal and spatiotemporal trends are described in subsequent sections.

#### Aggregated spatial patterns of wheat rust outbreaks

**Fig 1:**
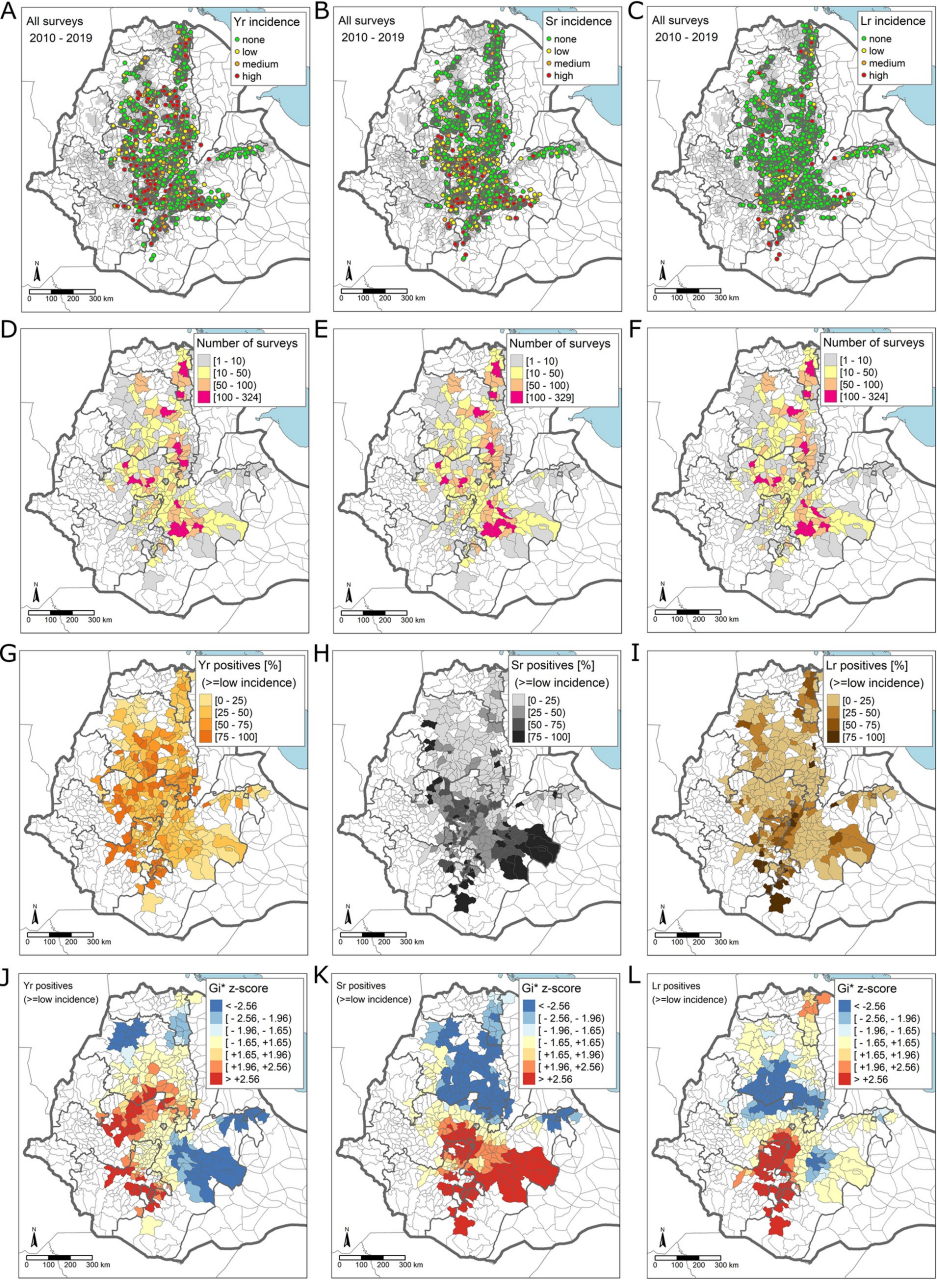
Spatial patterns of wheat rust outbreaks in Ethiopia in years 2010–2019. (left column) wheat stripe rust; (middle column) wheat stem rust; (right column) wheat leaf rust. In (**A-C**) all surveys of years 2010–2019 are mapped to illustrate the total spatial extent and disease patterns in point surveys. In (**D-F**) the number of surveys per administrative district are shown. (**G-I**) illustrates the proportion of positives per district (number of surveys with positive incidence values / total number of surveys). (**J-L**) shows hotspots with clustering of districts with high proportion of positives coloured in red and cold-spots with clustering of districts with low proportion of positives coloured in blue. Illustrated values are values of the Getis-Ord Gi* statistic. The Gi* statistic is a z-score indicating for each district if it belongs to a statistically significant cluster of districts with high (or low) proportions of positives. For example, values of Gi* > + 1.96 (or Gi* <– 1.96) indicate that the null-hypothesis of spatially random distribution of districts with high (or low) proportions of positives can be rejected at a significance level of 0.05 (1.96 standard deviations of the normal distribution). See S1–S4 Figs for a similar analysis using only moderate/high incidence scores as well as using severity scores. Maps created using R as GIS [18–22].

We mapped the point surveys from all years 2010–2019, aggregated them by administrative district and identified hot (high frequency of occurrence) and cold (low frequency of occurrence) spots of wheat rusts based on the average incidence (and severity) rates per district (Fig 1; see also S1–S4 Figs). Mapping all point surveys for each wheat rust (Fig 1A–1C) illustrates the wide area covered by the wheat rust surveillance programme with survey points in all Ethiopian wheat producing areas. Visual inspection of the distribution of low, moderate, and high incidence cases indicates that all three wheat rusts have occurred in almost all parts of Ethiopia. Counting the number of fields surveyed in each administrative district of Ethiopia shows that the distribution of wheat rust surveys varies substantially amongst districts (Fig 1D–1F). There are approximately 15 districts with higher numbers of fields surveyed (a total of approximately 100–300 field surveys are available from these districts for years 2010–2019). For most districts, around 10–100 field surveys are available. For districts at the periphery or far away from the main survey routes only small numbers of 1–10 field surveys from 10 years are available. The variations in the number of available field surveys with substantially higher number of field surveys (more than a factor of 10) in some districts and a lower number of field surveys in other districts needs to be considered when inferring national-scale trends based on the number of positive reports in a certain area. In areas with a substantially higher number of field surveys, the numbers of positive reports (disease: yes) can be expected to be higher than in other districts with similar production conditions simply because more fields were surveyed. To assess disease risk, we therefore map the proportion of positives per district (number of positives / total number of fields surveyed) for identifying where disease risk is highest.

The district-scale aggregation of the proportion of positives indicates a non-random spatial distribution of disease occurrence for all wheat rusts (Fig 1G–1I). Calculating the global *Morans-I* statistic confirms a statistically significant spatial autocorrelation in the proportion of positives per administrative district for all rusts (proportion of positives shown in Fig 1G–1I; *Morans-I* calculated on district scales; p-values <<0.05; details in S1 Appendix). A hotspot analysis (Getis-Ord Gi*) reveals pronounced hot and cold spots consisting of clusters of administrative districts with high (and low) proportion of positives (Fig 1J–1L; details in S1 Appendix). Very high proportions of stripe rust positives are clustered in south-western and central-western districts, indicating higher chances of finding stripe rust positives in these western/central parts of Ethiopia. The proportions of stripe rust positives in south-eastern districts is also moderate to high (Fig 1G). There is a pronounced south-north trend in the occurrence of wheat stem rust with higher probabilities of finding wheat stem rust in southern and south-eastern parts of Ethiopia. The longitudinal (west-east) trend in wheat stripe rust prevalence and the latitudinal trend (south-north) for wheat stem rust are further confirmed by calculating aggregated mean disease prevalence at a set of longitudes (and latitudes) in Ethiopia (see S5 and S6 Figs). Hotspots of wheat leaf rust occur in central-southern districts and in a small area in the north of Ethiopia.

The spatial clustering of high (and low) proportion of positives, as illustrated in Fig 1J–1L, is based on all positives, i.e. those with low, moderate and high incidence values. When considering only moderate and high incidence cases and when analysing reports of disease severity (instead of incidence) hot and cold spots can also be identified in geographic proximity to those shown here (compare Fig 1 with S1–S4 Figs). As the number of available surveys in some of the administrative districts of Ethiopia (e.g. the southernmost districts) is very low, results for these districts are associated with large uncertainties.

#### Long-term mean wheat rust disease prevalence on wheat fields at different altitudes

Wheat is grown in the Ethiopian highlands at various altitudes ranging from approximately 1500 above sea-level (m asl) to 3000 m asl and most field surveys analysed here were conducted between 2000 m asl and 3000 m asl. Analyses were conducted to test for associations between altitude and wheat rust prevalence for each of the three rusts.

**Fig 2:**
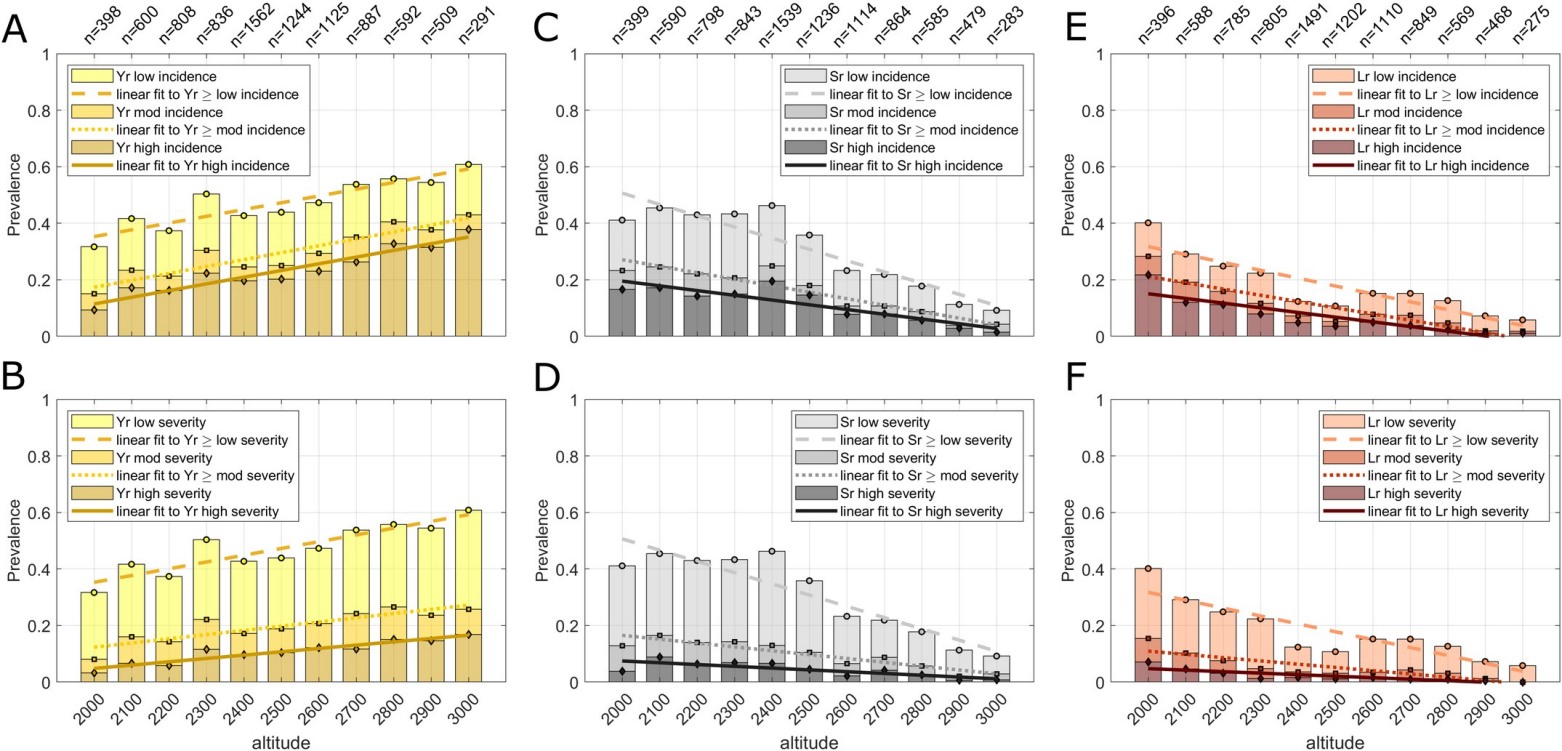
Linear correlation between altitude and wheat rust prevalence in Ethiopia. **(A-B)** wheat stripe rust; **(C-D)** wheat stem rust; **(E-F)** wheat leaf rust. The top row shows disease incidence, and the bottom row shows disease severity. The labels at the top of the x-axes show the total number of surveys per altitude bin. Prevalence is calculated as: number of surveys with disease incidence (severity) / total number of surveys per altitude bin. The stacked bar graphs show the parts of all positives with low, moderate, and high disease levels. For example, out of the total 291 surveys shown in the right bar in panel A, approximately 38% (110 surveys) are fields with high incidence, 5% (15 surveys) are fields with moderate incidence and 18% (52 surveys) are fields with low incidence. The remaining 114 surveys are negatives (not illustrated). The lines are linear fits to the mean prevalence values, separately calculated for the categories: ≥ low, ≥ moderate, and high disease. The lines are fitted to the stacked prevalence values. For example, the “linear fit to Yr ≥ low incidence” in panel A is obtained from all surveys with incidence scores “≥ low incidence”.

There is a linear dependence of rust prevalence on altitude for all three rusts with pronounced differences amongst rusts (Fig 2). Stripe rust prevalence increases, whereas the prevalence of stem rust and leaf rust (in terms of either disease incidence or disease severity) decreases with increasing altitudes. For yellow rust the prevalence of high incidence cases increases with increasing altitude, while the prevalence of moderate and low incidence cases, considered separately, remains approximately constant with altitude (Fig 2A). This is different for yellow rust severity, for which prevalence of high and low values increases with increasing altitudes (Fig 2B). For stem and leaf rust the prevalence in each disease level (low, mod, high) decreases with increasing altitudes (Fig 2C–2F). The altitude dependence is consistent with reported temperature ranges for the rusts: for example, stripe rust has lower reported temperature optima for growth and reproduction than stem rust [25]. The temperature at different altitudes in Ethiopia varies with region and month of the year, approximate long-term average ranges are 12–20°C at 2000 m asl and 6–14°C at 3000 m asl [27]. Likely, temperature is the core driver behind the observed trend in rust prevalence, but other factors, such as soil type and cultural practises, also play a role.

### Wheat rust prevalence on major wheat varieties in Ethiopia

**Fig 3:**
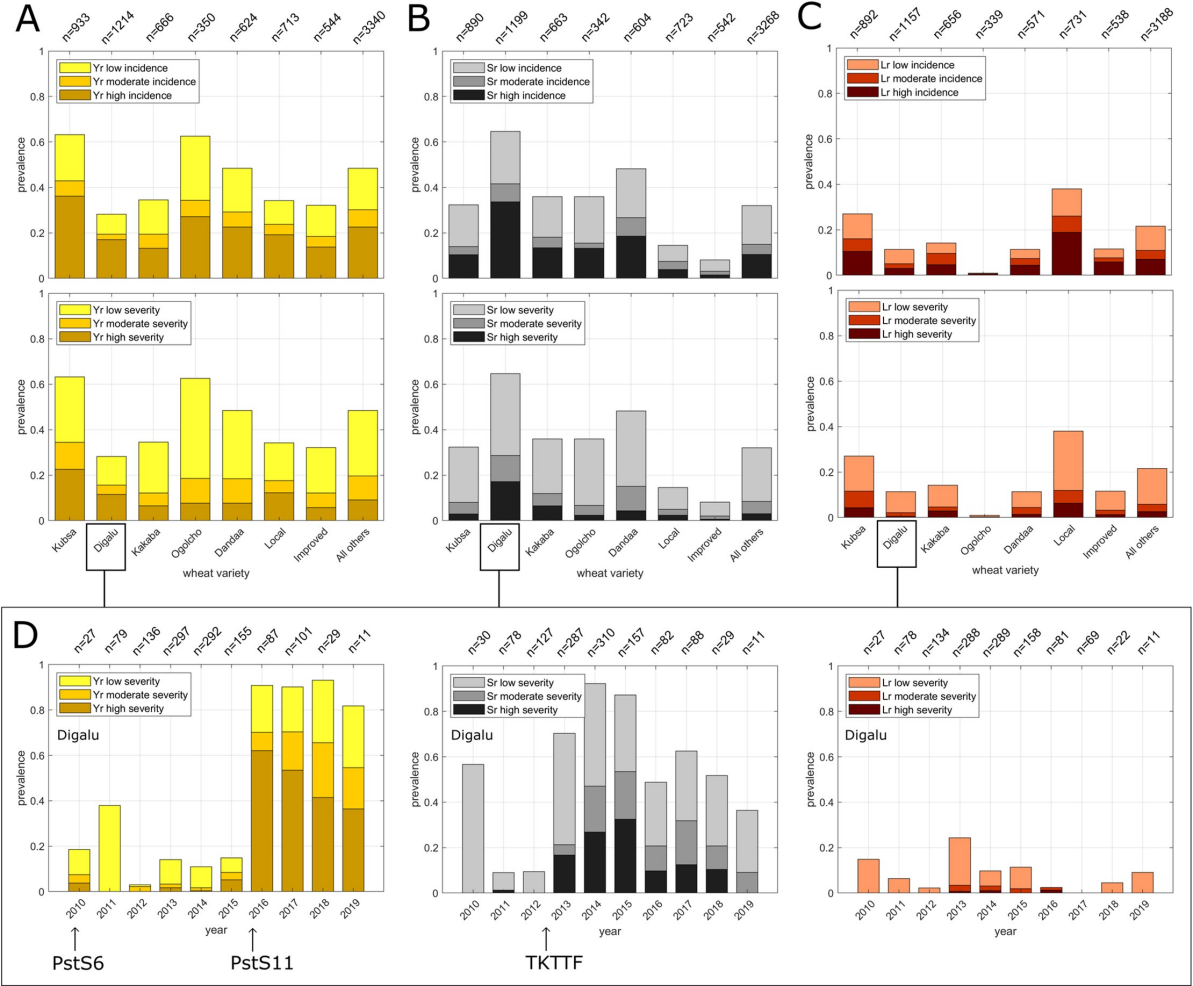
Wheat rust disease prevalence on major wheat varieties grown in Ethiopia. **(A)** wheat stripe rust; **(B)** wheat stem rust; **(C)** wheat leaf rust. (**A-C**) show the long-term mean disease prevalence during years 2010–2019 on key wheat varieties grown in Ethiopia (top: incidence; bottom: severity). (**D**) shows inter-annual variations of disease severity on the widely deployed wheat variety, *Digalu* (see S7 Fig for the corresponding incidence scores). The labels at the top of the x-axes show the total number of surveys per year. Prevalence is calculated as: number of surveys with disease incidence (severity) / total number of surveys per wheat variety. The years of first detection of wheat stripe rust pathogen races PstS6 [28,29] and PstS11 [30] and wheat stem rust pathogen races TKTTF [4] are illustrated at the bottom of (**D**).

The prevalence of wheat rusts differed significantly amongst wheat varieties (Fig 3A–3C; S7 Fig): stripe rust prevalence is highest on wheat variety *Kubsa* (highly susceptible to stripe rust since 2010), followed by *Ogolcho* and *Dandaa* (both susceptible to stripe rust since 2016); stem rust prevalence is highest on wheat variety *Digalu* (highly susceptible to stem rust since 2013), followed by *Dandaa* and *Ogolcho* (susceptible to stem rust since 2019); leaf rust prevalence is highest on *local* wheat varieties, followed by *Kubsa* and *Kakaba*. Chi-square-tests were conducted to test pairwise differences in the number of positives on different wheat varieties. For stripe rust this shows that disease prevalence (> = low) on *Kubsa* is significantly higher than on all other varieties (p-value<<0.05), except for *Ogolcho*, for which differences are not significant (p-value>0.5); for stem rust it shows that disease prevalence (> = low) is significantly higher on *Digalu* compared with all other wheat varieties (p-values<<0.05). Stem rust prevalence is lowest on varieties reported as “*improved*”, and significantly lower on varieties reported as “*local*” compared with *Digalu*, *Kubsa*, *Kakaba* and *Dandaa* (p-values<<0.05). It should be noted that, the broad categories “*local*” and “*improved*” are reported in surveys without further information about the specific variety and are associated with larger uncertainties, as they may encompass a wide range of species and varieties (including improved bread wheat varieties, tetraploid landraces, or occasionally also triticale and barley).

There are marked trends in the prevalence of rust on the widely grown wheat variety *Digalu* (Fig 3D; S7 Fig). For several years after the deployment of wheat variety *Digalu*, stripe rust prevalence was low (until 2015). In 2016, disease prevalence increased sharply to over 90% and stayed very high in subsequent years. The year of sharp increase in stripe rust prevalence on *Digalu* coincides with the year of first detection of stripe rust race PstS11 in Ethiopia [30]. Wheat stem rust prevalence is also low during the first years after deployment of *Digalu*. However, in 2013, the year after first detection of the new stem rust race TKTTF in Ethiopia, stem rust prevalence increased sharply and stayed high in subsequent years.

### Temporal analysis of wheat rust outbreaks in Ethiopia

For the analyses in this section field surveys from all locations were aggregated to focus exclusively on temporal patterns of wheat rust outbreaks across years from 2010 to 2019.

#### Inter-annual variations and trends across years

**Fig 4:**
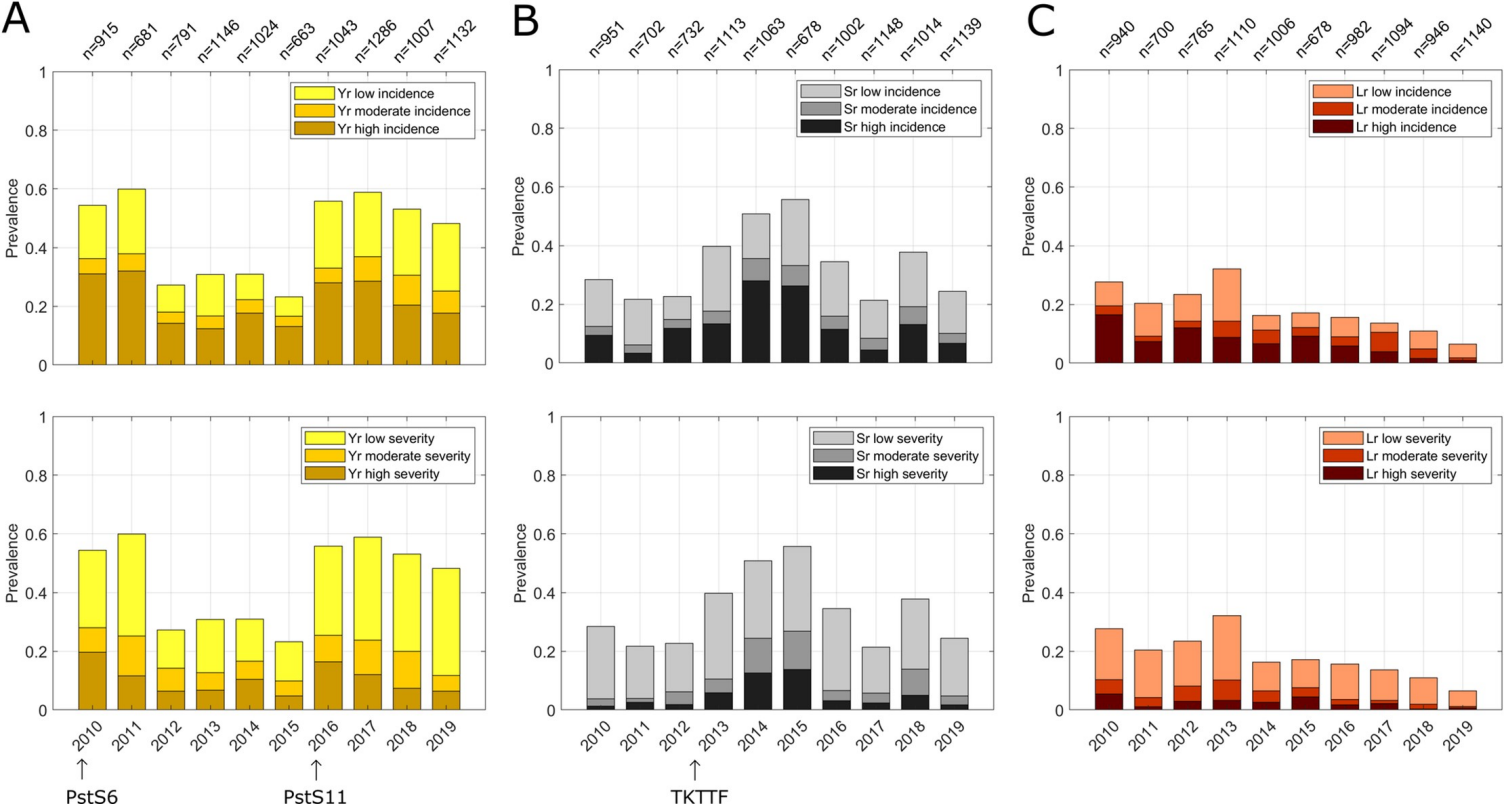
Inter-annual variations in aggregated total wheat rust prevalence in Ethiopia. **(A)** wheat stripe rust; **(B)** wheat stem rust; **(C)** wheat leaf rust. The top row shows prevalence of incidence scores and the bottom row shows prevalence of severity scores. The labels at the top of the x-axis show the total number of surveys per year. Prevalence is calculated as: number of surveys with disease incidence (severity) / total number of surveys per year. At the bottom, the years of first detection of wheat stripe rust pathogen races PstS6 [28,29] and PstS11 *[30]* and wheat stem rust pathogen race TKTTF [4] are illustrated.

Aggregating all surveys within each year reveals substantial variations amongst years in wheat rust outbreaks in Ethiopia (Fig 4). Peaks of stripe rust infection were detected in 2010 and 2011 and again in 2016–2018. Peaks of stem rust infection occurred in 2013–2015. Leaf rust prevalence was highest in 2010 and 2013. There is evidence of trends across years: for example, stem rust prevalence increases from 2011 until 2015 and then decreases in subsequent years. Also, in years with high stripe rust prevalence, stem rust is low (and vice-versa). There has been a linear decrease of leaf rust prevalence over time, but there is no evidence for comparable trends in stripe and stem rust prevalence (S8 Fig).

It seems likely that incursion of new pathogen races in combination with large areas planted to susceptible varieties accounts for some of the inter-annual variation in rust prevalence. Most infections of stripe rust during the peak year 2010 were caused by race PstS6 with Yr27 virulence [28,29] with large areas planted to highly susceptible varieties *Kubsa* and *Galema* and highly favourable weather conditions. In subsequent years, the wheat variety *Digalu*, resistant to race PstS6, was adopted on large scales throughout Ethiopia, which led to a decrease in stripe rust prevalence. However, in 2012 the wheat stem rust race, TKTTF, was first detected in Ethiopia, and *Digalu* was highly susceptible due to virulence on the resistance gene *SrTmp*, resulting in peak infections of stem rust in 2013 and 2014 [4]. In 2016, the stripe rust race PstS11 was detected, which caused most infections in subsequent years [30].

#### Within-season disease progress

**Fig 5:**
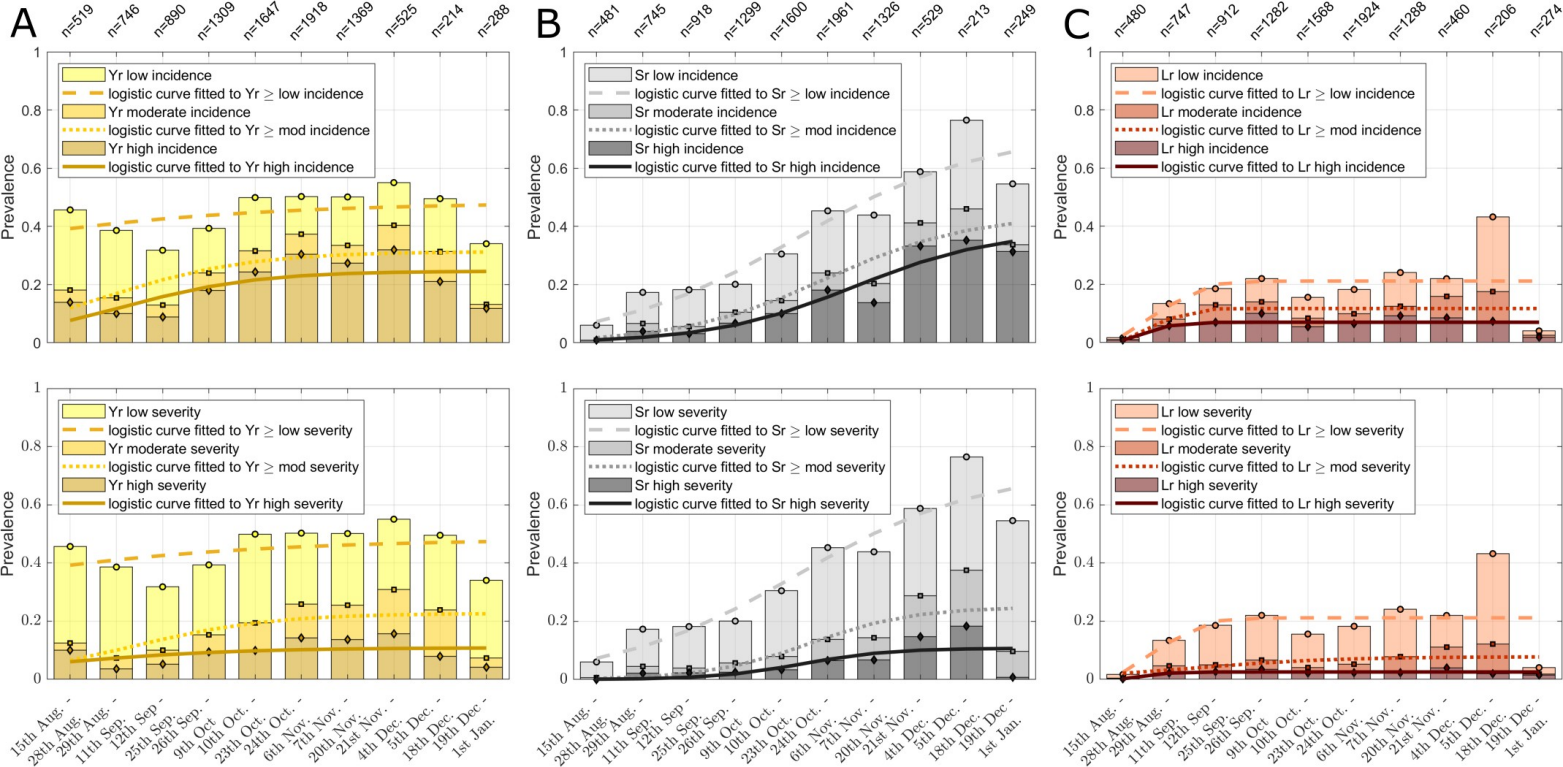
Long-term average within-season wheat rust disease progress in Ethiopia. **(A)** wheat stripe rust; **(B)** wheat stem rust; **(C)** wheat leaf rust. The time of the main wheat season in Ethiopia is separated into a set of bi-weekly time-intervals (x-axis). All surveys for years 2010–2019 are grouped into the bi-weekly time-intervals. The labels at the top of the x-axis show the total number of surveys per time-interval. For each time-interval, the mean disease prevalence (y-axis) is calculated (top row: incidence; bottom row: severity). The stacked bar graphs show the parts of all positives with low, moderate, and high disease levels. The lines show a logistic curve (see Eq 2) fitted to the mean prevalence data, separately calculated for the categories: ≥ low, ≥ moderate, and high disease. Note that there are substantial inter-annual variations (Fig 4) in disease prevalence, which are not shown in this illustration of the long-term average within-season disease-progress.

We aggregated surveys from all years into bi-weekly time-windows covering the main wheat season (August–December) for analysing within-season disease progress (Fig 5). Visual inspection shows that, for all three rusts, the prevalence (averaged over all years) increases during the first half of the main wheat season before reaching a plateau. Wheat stem rust shows the most pronounced increase with low levels at the beginning and high prevalence (> 50%) at the end of the season. The mean prevalence of wheat stripe rust increases strongest in months October and November. Leaf rust increases and levels off early in the season.

Standard disease progress models suggest a sigmoidal growth curve for polycyclic diseases such as rusts [31]. A logistic curve of the form

$$y(t)=\frac{\alpha }{1+exp(-\beta (t-\tau ))}$$ (2)

for modelling prevalence levels, *y*(*t*), at different times, *t*, of the main wheat season was fitted to the survey data (Fig 5). The coefficients, {*α,β,τ*}, obtained from a nonlinear fit conducted separately for each rust and disease score are summarized in S1 Table (obtained using the MATLAB function *fitnlm*). For wheat stem rust, the average within-season disease progress is described well by the logistic curve (Fig 5), while the other two rusts are only crudely approximated by Eq 2. Note that disease progress deviates markedly from the mean growth curve in some years, while in other years disease progress is close to the long-term average.

We focussed our analyses of within-season disease progress on the main wheat season because the aggregated total of approximately 400 surveys available for each rust for the minor wheat season (months April-July) is much lower than the aggregated total of approximately 10,000 surveys available for each rust for the main wheat season (months August-December). The aggregated total proportion of positives (with disease scores ≥ low) for all three rusts is substantially lower during the minor wheat season (< 20% for all rusts) compared with the main wheat season.

### Spatiotemporal analysis of wheat rust outbreaks in Ethiopia

#### Inter-annual variations in the spatial pattern of past wheat rust epidemics

The surveys of each year 2010–2019 were aggregated and mapped to provide temporal snapshots of epidemics in each year. Wheat rust incidence and severity scores from surveys were mapped separately for each rust. See S1–S6 Movies for time-lapse animations of yearly snapshots of disease severity and incidence cases for each of the three rusts.

There are substantial variations in the spatial patterns of stripe rust outbreaks over time (S1 and S2 Movies; also selected snapshots in S9 and S10 Figs). For example, in 2010 moderate and high stripe rust incidence cases are reported from a vast area covering the entire western and central wheat producing areas of Ethiopia. The total aggregated disease prevalence in 2010 was also substantially higher than in some other years (see Fig 4), confirming that 2010 was a major stripe rust epidemic year (as previously reported [5]). In 2014, there were substantially fewer stripe rust positives, and these are constrained to a smaller area in the north-west. Stem rust was widely distributed in southern and central areas in 2014 (see S3 and S4 Movies). Comparing the spatial distribution of stem rust and stripe rust indicates that in some years the geographic areas with higher stem rust prevalence were characterized by a lower stripe rust prevalence (and vice-versa). For example, in 2014 there are high incidence reports of wheat stem rust widely distributed in the south-eastern (Bale, Arsi zones) and central-western parts (Shewa zones) of Ethiopia, where wheat stripe rust reports are mostly negative. By contrast, in the northern areas of the country, stem rust reports in 2014 are mostly negative, but wheat stripe rust is reported with moderate and high incidence. However, clearly, both rusts also occur simultaneously in the same geographic areas.

#### Within-season spatiotemporal variations in past wheat rust outbreaks

For studying the within-season spatiotemporal patterns of past wheat rust outbreaks, a set of temporal snapshots of survey results was mapped at two-weekly intervals during the wheat seasons for 2010–2019. The incidence and severity scores were mapped separately for each wheat rust and visualized in time-lapse animations of within-season wheat rust outbreaks in Ethiopia during the last decade (S7–S12 Movies).

Few initial surveys are conducted early in the main wheat season with a much larger number of surveys occurring later, leading to many reports of wheat rust infections within a short time-interval at various geographical areas of Ethiopia (see S7, S9 and S11 Movies; also selected snapshots in S11 and S12 Figs). Two examples highlight the complexity of within-season spatiotemporal variations and trends in past wheat rust outbreaks: (i) in some regions and time-intervals, visual inspection of the data appears to suggest that an initially small outbreak in a certain region has grown over time with more high incidence cases reported in geographic proximity to the initial infections at a later time during the wheat season (see e.g. S11A Fig, comparing the wheat stripe rust outbreak in the north-western part of Ethiopia in the maps from left to right; also S9 and S10 Movies, stem rust outbreak in 2013); (ii) on other occasions, visual inspection of the data does not indicate where the outbreak might have started (see e.g. S11B Fig, left map; S9 and S10 Movies, year 2014). Further interpretation of these trends to infer the exact spatiotemporal characteristics of past outbreaks is limited by the limited number of available surveys (relative to all wheat fields on national scales) and variations in the selection of survey locations.

### Estimating financial losses due to wheat rusts

**Fig 6:**
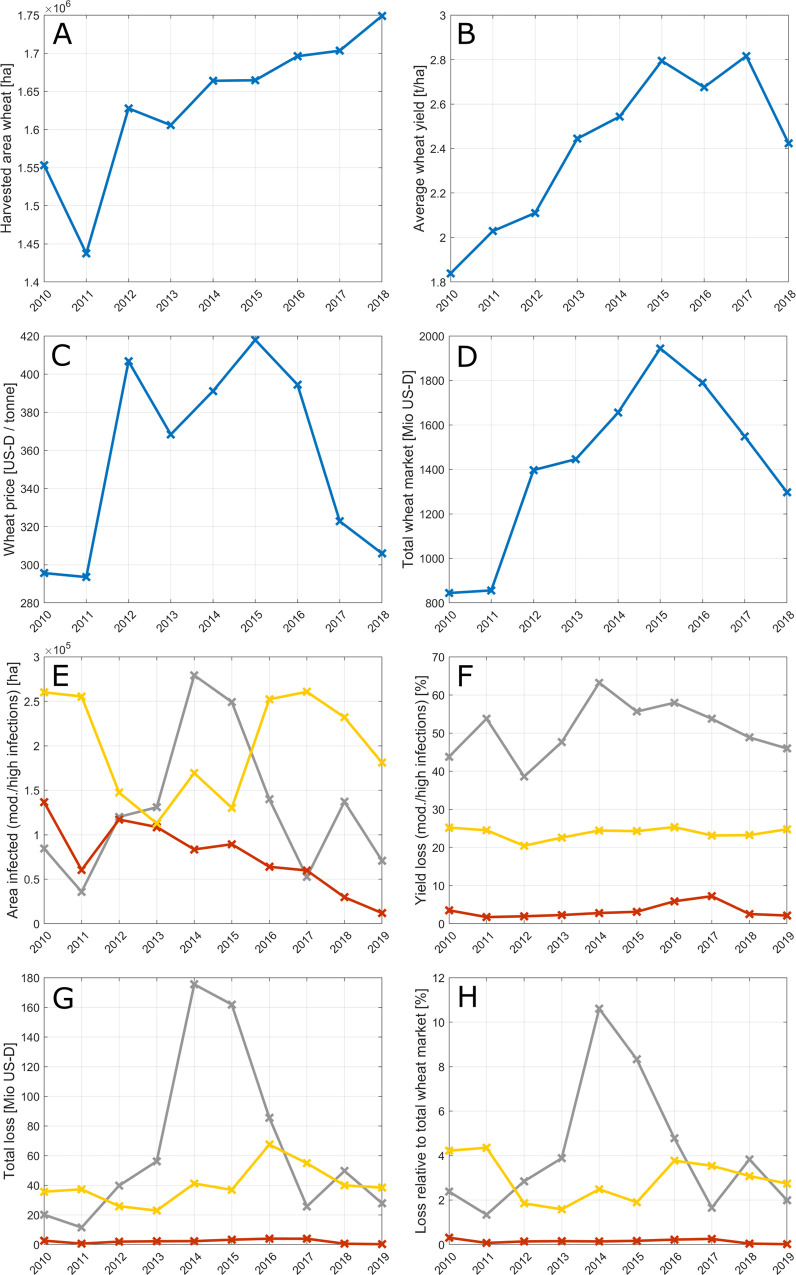
Wheat production in Ethiopia and estimated financial losses caused by wheat rust outbreaks during years 2010–2019. Blue: FAO data; grey: wheat stem rust; yellow: wheat stripe rust; red/brown: wheat leaf rust. (**A-D**) show national wheat production statistics of Ethiopia obtained from FAOSTAT [1]. (**E-H**) illustrate our estimates of the damage caused by wheat rusts during years 2010–2019. (**E**) shows the estimated area infected with wheat rusts; (**F**) shows the estimated fraction of yield lost due to wheat rusts; (**G**) shows the approximate total financial loss caused by wheat rusts; and (**H**) shows the approximate loss relative to the national total financial value of wheat produce at market price per year. As no FAO statistics were available for year 2019 at the time of this study (last checked on the 20^th^ of June 2020), we use the mean of years 2010–2018 as input for our estimates of yield losses in year 2019.

Estimates of annual losses (Eq 1) caused by wheat rusts in Ethiopia during years 2010–2019 are summarized in Fig 6, along with national-scale FAO statistics about wheat growing areas, average wheat yield, wheat price and the total financial value of wheat produce at market price. During the last decade, the area of wheat grown in Ethiopia increased from around 1.55 million ha in 2010 to around 1.75 million ha in 2018 (Fig 6A). There was also a notable increase in the average wheat yield from around 1.8 t/ha in 2010 up to 2.8 t/ha in 2017, dropping to 2.5 t/ha in 2018 (Fig 6B). Wheat prices have fluctuated between US-D 300 and US-D 400 per tonne (Fig 6C). The total financial value of wheat produce at market price in Ethiopia amounts to approximately US-D 1–2 billion per year with an increase in value over recent years, along with an increase in wheat area and wheat yield, modulated by wheat prices (Fig 6D).

We estimate that of the order of *A_rt_*(*i*)≈1−2×10^5^ hectares were moderately or highly infected with wheat rusts every year during 2010–2019 with differences between wheat rusts and substantial inter-annual variations (Fig 6E). For example, during years with large stripe rust outbreaks, as in 2010, our analyses suggest that approximately 250,000 ha were moderately or highly infected with wheat stripe rust. Our estimates of areas infected with wheat stem rust are highest in epidemic years around 2014. The fraction of yield lost to rusts (*Y_rt_* (*s, g*) in Eq 1) is highest for wheat stem rust, followed by wheat stripe rust and wheat leaf rust (Fig 6F). The maxima in the fraction of yield lost to disease coincide with years of strong outbreaks (e.g. stem rust, year 2014; stripe rust, year 2010).

Wheat rusts have caused substantial income losses, which we estimate on average to be of the order of 10s of millions US-D per year in the time-interval 2010–2019, with strong differences amongst the rusts and substantial inter-annual variations (Fig 6G). In relative terms, these losses correspond to approximately 2–10% of the total value of all wheat produced in Ethiopia (Fig 6H). Income losses caused by wheat stem rust are highest compared with the other rusts. Total loss estimates for stem rust vary between approximately US-D 170 million in years of strong outbreaks (peak in 2014) and approximately US-D 40 million in years with smaller outbreaks (minimum in 2011). During the years of highest losses from stem rust epidemics, we estimate that approximately 10% of the total value of wheat was lost to stem rust (Fig 6H). In relative terms, the highest losses caused by wheat stripe rust occurred in 2010 and 2011, when approximately 4% of the total value of wheat was lost to stripe rust (Fig 6G). This coincides with expectations about high losses in 2010, which was previously reported as a year with a major wheat rust outbreak [5]. The total loss of income (in million US-D) caused by wheat stripe rust is not highest in 2010, as might have been expected in terms of total areas infected, but it is higher in later years (peak in 2016), because of higher average wheat yields and higher wheat prices during those years, as well as larger total areas planted with wheat. Total loss estimates for stripe rust vary between approximately US-D 65 million (peak in 2016) and US-D 20 million in years with smaller outbreaks (minimum in 2013). Loss estimates for leaf rust are much lower and vary between approximately US-D 3.9 million (peak in 2017) and US-D 0.2 million (minimum in 2019). The assumptions underlying the analysis and appropriate caveats are reviewed in the Discussion.

### Simple models for predicting wheat rust outbreaks

In this section we test the performance of two simple empirical models for predicting wheat rust prevalence in Ethiopia. While the analysis of wheat rust survey data in previous sections has shown substantial variations and complexity of outbreak patterns during the last decade, we have also identified some pronounced spatiotemporal trends in wheat rust outbreaks. For example: the long-term mean within-season disease progress follows a sigmoidal type of growth-curve; there is a pronounced south-north trend in the occurrence of wheat stem rust; and there is clear linear correlation between altitude and disease prevalence for all three rusts. Some of these trends could in principle be reproduced by simple empirical models. Here we address the following questions: how well do simple empirical models perform in predicting wheat rust occurrence ahead of time—are they better than random choice? Can empirical models be useful for predicting disease occurrence and guiding surveillance and control measures?

We consider two simple examples of empirical models for wheat rust occurrence: *Model 1*—the time-dependent logistic curve defined in Eq 2, with parameters determined from fitting the model to the survey data; *Model 2*—a space- and time-dependent logistic curve defined as follows

$$Y^{\prime }(t,X,Y,Z)=\frac{1}{\left(1+\exp\left(\mu X+\nu Y+\rho Z+\delta t\right)\right)}$$ (3)

with independent variables time (*t*), latitude (*X*), longitude (*Y*), altitude (*Z)*, and parameters {*μ,ν,ρ,δ*} determined from fitting the model to survey data (separately for each rust and disease metric). Both models yield predictions in the form of a continuous risk score between 0 and 1 (Y in Eq 2, and *Y*′ in Eq 3).

The categorical data for disease status at each time and survey location were dichotomized into a binary score (diseased/not diseased). The survey data were separated into a training set comprising data from all but one of the survey years and with the data for the remaining year as a test set. The models with parameter values obtained from the fit to the training-data are used for predicting disease occurrence at all survey locations in the test-data. The analyses are repeated for ten different sets of training and test-data: each year (2010–2019) is used as test-data once. A receiver operating characteristic (ROC) curve analysis was used to test model performance, which includes calculating specificity and sensitivity values for different thresholds for binary classification of continuous risk scores obtained from the empirical models. The area under the curve (AUC) values and accuracy were calculated as performance metrics. The analyses were also repeated with an alternative dichotomization scheme classifying only moderate and high disease scores (considering incidence and severity separately) in surveys as “diseased” and all surveys with no or low disease scores as “healthy”.

**Fig 7:**
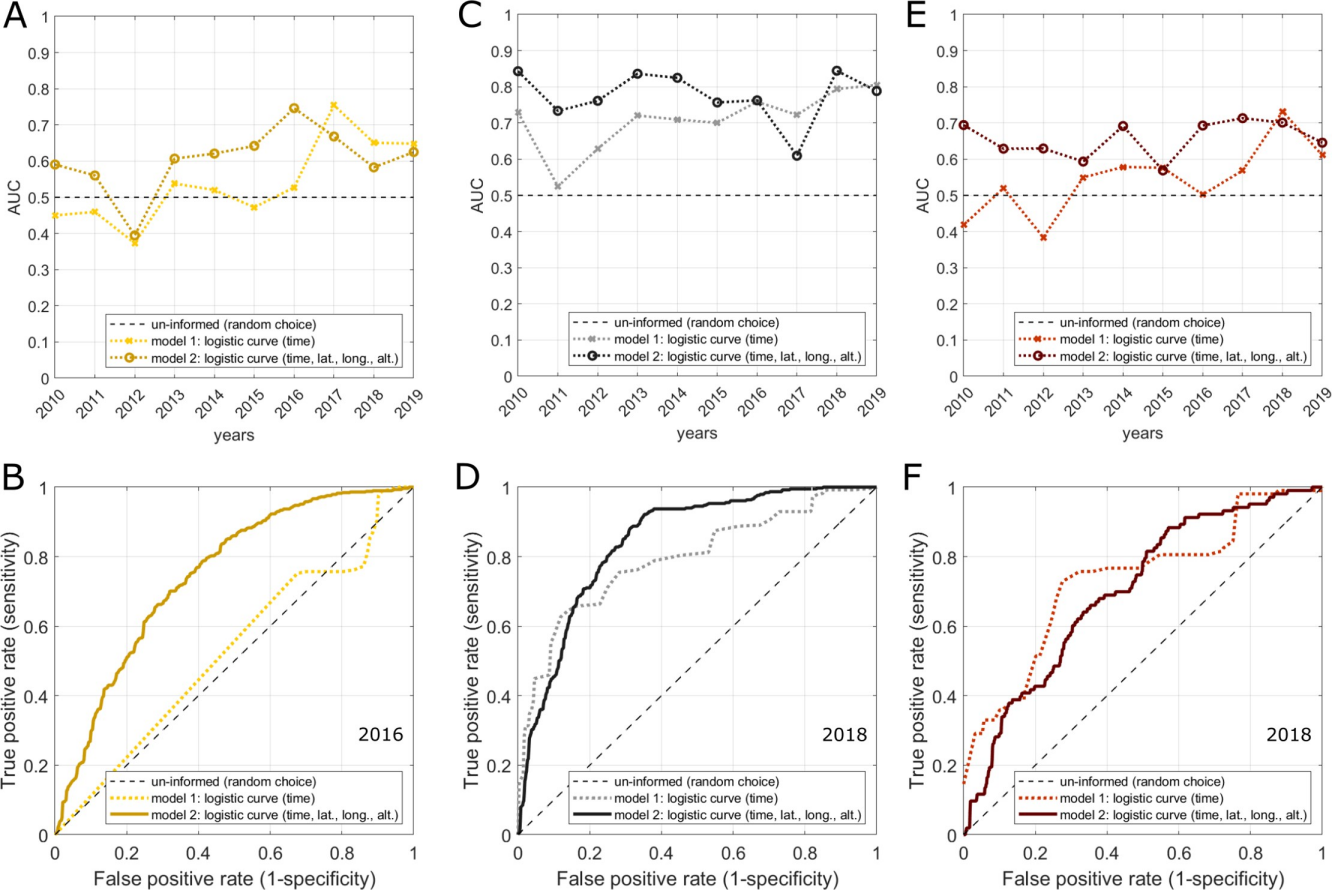
Performance of simple logistic models for predicting wheat rust occurrence in Ethiopia. **(A-B)** wheat stripe rust; **(C-D)** wheat stem rust; **(E-F)** wheat leaf rust. Two simple logistic models were used to predict wheat rust occurrence: a temporal model (model 1, see Eq 2) predicting wheat rust occurrence as a function of the time since the start of the main wheat season and a spatiotemporal model (model 2, see Eq 3), predicting wheat rust occurrence as a function of the time since the start of the main season and the location in Ethiopia (latitude, longitude, and altitude). Model performance was tested by fitting the models to training data from all but 1 year of surveys and then conducting a ROC analysis for testing the performance of the fitted model against the data from the year not used for fitting (repeated for every year). The upper row shows the resulting AUC score of both models for each year and all rusts. The bottom row shows the corresponding ROC curves of one exemplar year. For the analysis illustrated here all survey entries with non-zero disease incidence were classified as “diseased” and all surveys with zero incidence were classified as “healthy”. The testing procedure was also conducted using an alternative dichotomization scheme classifying all surveys with moderate or high incidence values as “diseased” and all surveys with zero or low incidence as “healthy” (see S13 Fig).

Both Model 1 and Model 2 perform substantially better in predicting wheat stem rust than in predicting wheat stripe rust and leaf rust (Fig 7; and S2 Table, S13 Fig). For wheat stem rust, the mean AUC value (averaged over all test-years) for Model 1 (Eq 2) is 0.71, while Model 2 (Eq 3) achieves a mean AUC value of 0.78 (with accuracy of 71%). In years of strong stem rust epidemics, such as 2013, high AUC values of 0.84 are achieved. Model 2 predicts disease status at all survey points in year 2013 with an accuracy of 77% (and Model 1 with an accuracy of 67%). The AUC value is substantially better than random choice for every year of testing (2010–2019) (Fig 7). For stripe rust, Model 1 (Eq 2) performs very poorly, achieving a mean AUC value of 0.52, which is very close to what would be expected by random chance. Model 2 performs slightly better, but still poorly, with a mean AUC score of 0.6. Simple models for reproducing mean temporal and spatial trends in past outbreak patterns are not well suited for predicting stripe rust, because the mean trends in stripe rust outbreaks are less pronounced than the trends for stem rust occurrence and inter-annual variations for stripe rust are large. Model 1 (Eq 2) also performs very poorly for leaf rust, achieving a mean AUC value of 0.54. Model 2 performs better, with a mean AUC score of 0.66.

Comparing the performance of the two models in predicting different levels of disease (low, moderate, and high disease reports) shows slightly better performance in predicting strong infections compared with predicting all infections (see S2 Table, SI). Overall, the performance of the two simple models tested here is moderate for stem rust and poor for stripe and leaf rust. Importantly, the performance varies substantially amongst years. This is intuitive as the performance of the two empirical models depends on the degree of similarity in the outbreak pattern of the test-year and the mean outbreak pattern in all training-years. Using simple empirical models of the type introduced here for predicting wheat rusts ahead of time relies on the assumption that past outbreak patterns can be extrapolated into the future. Our results indicate that this may be a useful first approximation for stem rust: simple models for stem rust are moderately effective in predicting disease in epidemic years and do perform substantially better than random choice during all years for 2010–2019. However, for the other rusts the two models perform very poorly. Clearly, also for stem rust, simple models have a limited value, because they cannot account for any extreme events in mechanistic drivers of epidemics and are not suited well for including survey information in real-time into the modelling framework. More complex stochastic mechanistic models are needed and currently in use for predicting wheat rusts in Ethiopia [10]. The simple empirical models introduced here provide a useful baseline for comparison with complex predictive models based on coupling weather driven Lagrangian spore dispersion with environmental suitability models for infection.

## Discussion

Our analyses indicate that there were repeated wheat rust disease outbreaks in Ethiopia during the last decade, despite ongoing efforts for surveillance and control. This highlights the challenges to wheat production posed by wheat rusts in the highlands of Eastern Africa.

The key results for wheat rust outbreaks in Ethiopia include the following: there are substantial spatial differences in outbreak patterns of rusts in Ethiopia, with a marked north-south trend in the occurrence of stem rust and a west-east trend for stripe rust (Fig 1). Disease hotspots for stem rust occur in wheat producing areas south of Addis Ababa and east of the Rift Valley and for stripe rust in the central western wheat producing areas (Fig 1). Mean disease prevalence is linearly correlated with altitude for all three rusts (stem, stripe, and leaf). This information could be used to improve future control strategies by altitude-dependent deployment of rust resistant varieties (as has been considered previously; *pers*. *comm*., B. Hundie). Disease prevalence differs amongst wheat varieties (Fig 3) with evidence of boom-and-bust cycles on key varieties. Substantial temporal variations occur within and between years for both stem and stripe rust; years of peak prevalence of stripe rust correspond with lower prevalence of stem rust and vice versa (Fig 4); the prevalence of leaf rust declined over the decade, but for stripe rust and stem rust prevalence no significant change in mean prevalence occurred.

The differences in disease prevalence amongst wheat varieties (Fig 3) are likely to have been influenced by variety resistance gene composition, area planted and prevailing rust races. The survey data provide evidence of a strong influence of the incursion of new pathogen races on national-scale disease prevalence. The wheat variety *Kubsa*, originally released in 1995, was the predominant wheat variety grown in Ethiopia occupying over 30% of the wheat area in 2009/10 and extremely popular with farmers [32]. It, and another widely planted variety, *Galema*, became highly susceptible to stripe rust in 2010 due to the presence of race PstS6 of the pathogen with Yr27 virulence. *Kubsa* was widely replaced by *Digalu* after 2010 due to stripe rust susceptibility [32]. *Digalu*, released in 2005, increased rapidly in area after 2010 due to resistance to the PstS6 race of the stripe rust pathogen and resistance to the Ug99 race (TTKSK) of the stem rust pathogen. In 2013/14 *Digalu* was estimated to occupy at least 27% of the wheat area [32]. It became highly susceptible to stem rust race TKTTF in 2013 and subsequently highly susceptible to stripe rust race PstS11 in 2016. High susceptibility to both stripe and stem rust is now resulting in declining areas being grown to *Digalu*.

The temporal trends in disease prevalence on *Digalu* is an example of a boom-and-bust cycle of major gene resistance. This underlines two disadvantages of planting large monocultures of wheat varieties, such as *Digalu*, with major gene resistance to a specific pathogen: (i) while resistance to a certain pathogen or pathogen race may be ensured initially (as for stripe rust), susceptibility to other pathogens and novel races is a considerable risk (*Digalu* was highly susceptible to race TKTTF of stem rust due to the presence of the *SrTmp* resistance gene); (ii) there is a high risk that resistance will eventually break down, leaving large areas highly susceptible; in this case *Digalu* provided around four years of resistance to stripe rust after widespread adoption but then resistance broke down completely.

Leaf rust is the least prevalent, and the least economically damaging, of the wheat rusts in Ethiopia. Ethiopia has genetically divergent pathotypes of leaf rust [33], some of which are preferentially found on tetraploid wheat (durum and emmer wheats). This may account for the relatively high prevalence of leaf rust observed on varieties reported as ‘*local*’, as some tetraploid wheats would be included in this classification category. Presence of tetraploid wheats in the generic ‘*local*’ category could also contribute to lower prevalence of stem rust due to different resistance gene combinations; however, it should be noted that other cereals, e.g. barley and triticale, may have also been classified as ‘*local*’ by surveyors. While the widely deployed varieties, such as *Digalu*, are well-known and hence easier to identify by visual inspection during surveys, the correct identification of other varieties can be more difficult and requires genetic data. As this is not available for the current study, it remains unclear which varieties were reported as ‘*local*’ and ‘*improved*’ and what caused the differences in the number of positives reported for the generic categories ‘*improved*’ and ‘*local*’ in comparison with the most widely deployed wheat varieties (Fig 3).

To our knowledge, the estimates of economic losses caused by wheat rusts described in the results provide the most detailed approximations for Ethiopia, considering 10 years of survey data in combination with national-scale FAO statistics and published empirical relations between growth stage, disease severity and yield loss. The estimates, however, need to be interpreted as crude approximations associated with large uncertainties. Our loss assessment for the stripe rust outbreak in 2010 may be an underestimate, because our estimate for the infected area in 2010 is lower than previous estimates of infected areas in 2010 (in [5] it is stated that at least 400,000 ha were infected and [6] write that 600,000 ha were affected); however, the exact methodology used to arrive at previously stated estimates [5,6] is not available to us, making it impossible to compare estimates in detail. Here we only consider moderately and highly infected areas and explicitly take account of wheat prices, total wheat area and wheat yield. Large uncertainties remain, because only a small fraction of the total wheat area in Ethiopia was surveyed; yield losses depend on wheat variety and pathogen races in each wheat field, as well as on fungicide use-patterns and other management practises, for which data are available for some survey sites but not on national scales.

Survey locations are selected in part for early detection of rust outbreaks. This has the advantage that outbreaks detected early in the season can be used to obtain near-real time wheat rust risk assessments in the Wheat Rust Early Warning System in Ethiopia, for example by using disease detection sites as source-locations for long-range dispersal simulations [10]. Choosing different survey locations at different times of the year, however, has the disadvantage that, there are entire regions of Ethiopia for which no survey information is available early in the season (S11A–S11C Fig). If rust is detected later during the wheat season in these areas, then it is very difficult to infer if these were caused by previously undetected infections or by incursions from outbreaks in other areas of Ethiopia. To improve the understanding of national-scale outbreak patterns, it is essential to define a set of representative locations that are repeatedly surveyed at regular time-intervals in addition to the flexible surveys conducted for early detection.

If wheat rust epidemiology in Ethiopia is best characterized by a small number of strong infection sources early in the season that cause a large number of secondary infections via long-range dispersal and if all these strong initial infections are correctly detected early in the season, then the strategy of using early detection sites as key input for near-real time wheat rust disease warnings promises useful risk estimates. However, if wheat rust epidemiology in Ethiopia is best characterized by a large number of independent small outbreaks at various locations early in the season, and these remain undetected due to limited survey resources early in the season, then risk estimates that depend on the location of the initial detections (e.g. long-range dispersal simulations) may be misleading by showing some areas at risk, but not representing the major infection risks in the country. This is an area of active study.

Our analyses indicate that every year there are many independent outbreaks in various geographic areas of Ethiopia. However, there is also some evidence of local wave-like spread from an initial outbreak site (e.g. during the wheat stem rust outbreak in 2013; S9 and S10 Movies). Given the sparsity of the surveillance data, local scale dynamics involving the initiation and interaction of seemingly independent outbreak sites are as yet unclear. Previous experimental studies in Ethiopia involving spore traps confirmed that wheat rust urediospores are present in air year around [34,35]. Future work needs to consider the spatial and temporal fluctuations in environmental conditions favourable for spore release and infection, the mixing influence of airborne dispersal and influx of rust spores into Ethiopia [36,37], fungicide use (which was not reported in the survey data analysed here) and varietal diversification across the agricultural landscape of Ethiopia, characterized by many small farms.

The long-term surveillance data used in this study clearly highlights some geographical hotspots within Ethiopia, notably for stem and stripe rust. The key drivers causing these disease hotspots are unknown, but likely to be a combination of suitable environment (moisture, temperature) and the presence of susceptible hosts. Prior, large-scale epidemics are likely contributing factors through the high prevalence of positive disease reports. For stripe rust, the identified hotspot closely matches the worst affected areas of the 2010 epidemic. In these regions, large areas of highly susceptible varieties (*Kubsa* and *Galema*) were grown. For stem rust, the hotspot corresponds with the area affected by the 2013 epidemic and the main areas planted to the susceptible variety *Digalu*. Additionally, some preliminary results for stem rust indicate the prevalence of optimal elevation ranges (a surrogate for optimal temperatures) and clay rich soils (e.g., vertisols—that have high water holding capacity and hence high moisture content later in the growing season) in the southern half of the country. These apparently conducive environmental conditions also closely align with the identified hotspot.

Simple empirical models to account for disease prevalence at survey locations showed some promise for stem rust but failed for stripe and leaf rust. The performance of the simple empirical models introduced here is comparable to a previous study for classifying diseased and non-diseased wheat fields in Ethiopia based on a complex deep learning framework and satellite imagery [38]. The analyses of the survey data summarized in the current paper provide a starting point for ongoing work to test more mechanistic meteorological and epidemiological models that are integrated into an early warning system for wheat rusts in Ethiopia [10]. One test involves assessing how well the integrated mechanistic models reproduce the empirical characteristics of past wheat rust outbreaks in Ethiopia, as described in this paper. Other lines of research emerging from the empirical analyses reported here concern the following questions: are inter-annual variations in wheat stem rust and wheat stripe rust prevalence driven by large-scale cyclic variations in weather conditions (e.g. caused by El-Nino)? Which underlying environmental factors are the predominant cause for the linear correlation between altitude and disease prevalence (e.g. temperature, humidity, soil type, soil water content) or is this trend caused by different management practises at different altitudes? Do wheat stem rust infections start in the southern areas of Ethiopia, East of the Rift-Valley, and then spread northwards and westwards over the Rift-Valley as the main wheat season progresses, or are the infections observed in these different regions of Ethiopia independent of each other and caused by spread from volunteer plants or airborne pathogens from different sources?

The *RustTracker* dataset is a unique resource for studying wheat rust dynamics not only in Ethiopia. The complete dataset comprises survey data from surveys in more than 35 countries. This extraordinary data is available publicly for future research and for guiding surveillance and control measures. The methods for automated spatial data analysis and visualization, geographical mapping and statistical modelling used for the current study may be adapted for future analyses of rust outbreaks in other geographical areas.

## Supporting information

S1 Fig Spatial patterns of wheat rust outbreaks in Ethiopia in years 2010–2019 (moderate and high incidence cases). **(A)** stripe rust, **(B)** wheat stem rust, **(C)** wheat leaf rust. **(A-C: top row):** proportion of moderate rust incidence cases (number of surveys with moderate or high incidence scores / total number of surveys) per district; **(A-C: bottom row)** hot- and cold-spots with respect to the proportion of moderate or high rust incidence cases per district. **(D)** stripe rust, **(E)** wheat stem rust, **(F)** wheat leaf rust. **(D-F: top row):** proportion of high rust incidence cases (number of surveys with high incidence scores / total number of surveys) per district; **(D-F: bottom row)** hot- and cold-spots with respect to the proportion of high rust incidence cases per district. Maps created using R as GIS [18–22].
(DOCX)

S2 Fig Spatial patterns of wheat stripe rust outbreaks in Ethiopia in years 2010–2019 (severity scores). **(top row)** Proportion of low (left map), moderate (centre map) and high (right map) severity cases per district (calculated as: [number of surveys with disease severity score x / total number of surveys per district]). **(bottom row)** hot- and cold-spots of districts with high proportions of low (left map), moderate (centre map) and high (right map) stripe rust severity cases. Maps created using R as GIS [18–22].
(DOCX)

S3 Fig Spatial patterns of wheat stem rust outbreaks in Ethiopia in years 2010–2019 (severity scores). **(top row)** Proportion of low (left map), moderate (centre map) and high (right map) severity cases per district (calculated as: [number of surveys with disease severity score x / total number of surveys per district]). **(bottom row)** hot- and cold-spots of districts with high proportions of low (left map), moderate (centre map) and high (right map) stem rust severity cases. Maps created using R as GIS [18–22].
(DOCX)

S4 Fig Spatial patterns of wheat leaf rust outbreaks in Ethiopia in years 2010–2019 (severity scores). **(top row)** Proportion of low (left map), moderate (centre map) and high (right map) severity cases per district (calculated as: [number of surveys with disease severity score x / total number of surveys per district]). **(bottom row)** hot- and cold-spots of districts with high proportions of low (left map), moderate (centre map) and high (right map) leaf rust severity cases. Maps created using R as GIS [18–22].
(DOCX)

S5 Fig Latitudinal trends in wheat rust prevalence in Ethiopia in years 2010–2019. **(A)** wheat stripe rust; **(B)** wheat stem rust; **(C)** wheat leaf rust. A set of latitude-intervals are defined covering all latitudes at which wheat is grown in Ethiopia (x-axis). The surveys from all years are grouped according to their latitude coordinate and the aggregated mean prevalence is calculated for each latitude interval. (top row) long-term mean prevalence of incidence scores per latitude, (bottom row) long-term mean prevalence of severity scores per latitude. The labels at the top of the x-axes show the total number of surveys per latitude bin. Prevalence is calculated as: [number of surveys with incidence score x / total number of surveys per altitude-interval]).
(DOCX)

S6 Fig Longitudinal trends in wheat rust prevalence in Ethiopia in years 2010–2019. **(A)** wheat stripe rust; **(B)** wheat stem rust; **(C)** wheat leaf rust. A set of longitude-intervals are defined covering all longitudes at which wheat is grown in Ethiopia (x-axis). The surveys from all years are grouped according to their longitude coordinate and the aggregated mean prevalence is calculated for each longitude interval. (top row) long-term mean prevalence of incidence scores per longitude, (bottom row) long-term mean prevalence of severity scores per longitude. The labels at the top of the x-axes show the total number of surveys per latitude bin. Prevalence is calculated as: [number of surveys with incidence score x / total number of surveys per altitude-interval]).
(DOCX)

S7 Fig Interannual variations of disease prevalence (incidence scores) on wheat variety *Digalu*. **(A)** wheat stripe rust; **(B)** wheat stem rust; **(C)** wheat leaf rust. The labels at the top of the x-axes show the total number of surveys per year. Prevalence is calculated as: [number of surveys with disease incidence (severity) / total number of surveys per wheat variety].
(DOCX)

S8 Fig Assessing linear trends in interannual variations of wheat rust prevalence in Ethiopia in years 2010–2019. **(A)** wheat stripe rust; **(B)** wheat stem rust; **(C)** wheat leaf rust. Symbols (circle, square, diamond) show sample mean prevalence levels in surveys and lines are linear fits to the mean prevalence levels (obtained using the MATLAB function *fitlm*). For each wheat rust and each disease metric (low incidence, moderate incidence, high incidence, low severity, moderate severity and high severity) it was tested if the slope of the fitted linear model is different from a constant line with a slope coefficient of zero using the MATLAB function *CoefTest*(). According to this test there is no statistically significant decrease or increase in the mean wheat stripe rust and wheat stem rust disease prevalence during years 2010–2019. The resulting p-values for the F-Test that the slope coefficient of the linear model is different to zero are larger than a significance level of 0.05 for all linear models of stripe rust and stem rust. However, there is a statistically significant decrease of wheat leaf rust prevalence over the years. The p-values for the F-Test of the slope of all leaf rust models are all smaller than 0.05 except for the linear fit to high severity levels.
(DOCX)

S9 Fig Interannual variations in the spatial patterns of wheat rust outbreaks in Ethiopia (incidence scores). **(A)** wheat stripe rust; **(B)** wheat stem rust; **(C)** wheat leaf rust. The maps show disease incidence at all survey points of three exemplar years: 2010, 2014 and 2019. Symbols: green—no disease; yellow—low incidence; orange—moderate incidence; red—high incidence; grey areas—wheat producing regions. In 2010 a major wheat stripe rust epidemic led to infections covering large parts of western and central Ethiopian wheat producing areas (left map, A). In these regions, wheat stem rust incidence was low in 2010 (left map, B). In 2014 a major wheat stem rust epidemic occurred in large parts of southern and central Ethiopia (central map, B). In these areas wheat stripe rust incidence was low (central map, A). See S10 Fig for the corresponding severity scores at survey locations illustrated here. Maps created using R as GIS [18–22].
(DOCX)

S10 Fig Interannual variations in the spatial patterns of wheat rust outbreaks in Ethiopia (severity scores). **(A)** wheat stripe rust; **(B)** wheat stem rust; **(C)** wheat leaf rust. The maps show disease severity at all survey points of three exemplar years: 2010, 2014 and 2019. Symbols: green—no disease; yellow—low incidence; orange—moderate incidence; red—high incidence; grey areas—wheat producing regions. Maps created using R as GIS [18–22].
(DOCX)

S11 Fig Within-season variations in spatial patterns of wheat rust outbreaks in Ethiopia (incidence scores). The maps show disease incidence at all survey points at three different times of the main wheat season. Symbols: green—no disease; yellow—low incidence; orange—moderate incidence; red—high incidence; grey areas—wheat producing regions. **(A)** wheat stripe rust incidence at the beginning (left map), middle (centre map) and end (right map) of the main wheat season 2010; **(B)** wheat stem rust incidence at the beginning (left map), middle (centre map) and end (right map) of the main wheat season 2014; **(C)** wheat leaf rust incidence at the beginning (left map), middle (centre map) and end (right map) of the main wheat season 2010. See S12 Fig for the corresponding severity scores at survey locations illustrated here. Maps created using R as GIS [18–22].
(DOCX)

S12 Fig Within-season variations in spatial patterns of wheat rust outbreaks in Ethiopia (severity scores). The maps show disease severity at all survey points at three different times of the main wheat season. Symbols: green—no disease; yellow—low incidence; orange—moderate incidence; red—high incidence; grey areas—wheat producing regions. **(A)** wheat stripe rust severity at the beginning (left map), middle (centre map) and end (right map) of the main wheat season 2010; **(B)** wheat stem rust severity at the beginning (left map), middle (centre map) and end (right map) of the main wheat season 2014; **(C)** wheat leaf rust severity at the beginning (left map), middle (centre map) and end (right map) of the main wheat season 2010. Maps created using R as GIS [18–22].
(DOCX)

S13 Fig Performance of simple logistic models for predicting wheat rust occurrence in Ethiopia (moderate or high incidence cases). **(A)** wheat stripe rust; **(B)** wheat stem rust; **(C)** wheat leaf rust. Two simple logistic models were used to predict moderate or high wheat rust incidence cases: A temporal model (Model 1, see Eq 2 in the main text) predicting wheat rust occurrence as a function of the time since the start of the main wheat season and a spatiotemporal model (Model 2, see Eq 3 in the main text), predicting wheat rust occurrence as a function of the time since the start of the main season and the location in Ethiopia (latitude, longitude and altitude). Model performance was tested by fitting the models to training data from all but 1 year of surveys and then conducting a ROC analysis for testing the performance of the fitted model against the data from the year not used for fitting (repeated for every year). The upper row shows the resulting AUC score of both models for each year and all rusts. The bottom row shows the corresponding ROC curves of one exemplar year. For example, the ROC curve of wheat stem rust (centre graph, bottom row) for the test-year 2013 corresponds to the AUC value of the model for wheat stem rust in year 2013 illustrated in the graph at the centre of the top row. For the analysis, all survey entries with moderate or high incidence values as “diseased” and all surveys with zero or low incidence as “healthy”.
(DOCX)

S1 Table Parameter estimates for the univariate logistic model for wheat rust disease progress. (DOCX)

S2 Table Performance of simple empirical models for wheat rusts in Ethiopia (performance metrics). (DOCX)

S1 Movie Interannual variations of wheat stripe rust outbreaks in Ethiopia (incidence scores). (MP4)

S2 Movie Interannual variations of wheat stripe rust outbreaks in Ethiopia (severity scores). (MP4)

S3 Movie Interannual variations of wheat stem rust outbreaks in Ethiopia (incidence scores). (MP4)

S4 Movie Interannual variations of wheat stem rust outbreaks in Ethiopia (severity scores). (MP4)

S5 Movie Interannual variations of wheat leaf rust outbreaks in Ethiopia (incidence scores). (MP4)

S6 Movie Interannual variations of wheat leaf rust outbreaks in Ethiopia (severity scores). (MP4)

S7 Movie Within-season variations of wheat stripe rust outbreaks in Ethiopia (incidence scores). (MP4)

S8 Movie Within-season variations of wheat stripe rust outbreaks in Ethiopia (severity scores). (MP4)

S9 Movie Within-season variations of wheat stripe rust outbreaks in Ethiopia (incidence scores). (MP4)

S10 Movie Within-season variations of wheat stripe rust outbreaks in Ethiopia (severity scores). (MP4)

S11 Movie Within-season variations of wheat stripe rust outbreaks in Ethiopia (incidence scores). (MP4)

S12 Movie Within-season variations of wheat stripe rust outbreaks in Ethiopia (severity scores). (MP4)

S1 Appendix Spatial analysis of wheat rust outbreaks in Ethiopia. (DOCX)

S2 Appendix Estimation of financial losses caused by wheat rusts in Ethiopia. (DOCX)
