# Supplementary material for: Wheat rust epidemics damage Ethiopian wheat production: A decade of field disease surveillance reveals national-scale trends in past outbreaks
Source: PLoS One. 2021 Feb 3;16(2):e0245697. doi: 10.1371/journal.pone.0245697 (PMC7857641; doi:10.1371/journal.pone.0245697)
Supplement: S1 Fig — (A) stripe rust, (B) wheat stem rust, (C) wheat leaf rust. (A-C: top row): proportion of moderate rust incidence cases (number of surveys with moderate or high incidence scores / total number of surveys) per district; (A-C: bottom row) hot- and cold-spots with respect to the proportion of moderate or high rust incidence cases per district. (D) stripe rust, (E) wheat stem rust, (F) wheat leaf rust. (D-F: top row): proportion of high rust incidence cases (number of surveys with high incidence scores / total number of surveys) per district; (D-F: bottom row) hot- and cold-spots with respect to the proportion of high rust incidence cases per district. Maps created using R as GIS [18–22]. (DOCX) [file pone.0245697.s001.docx]

**
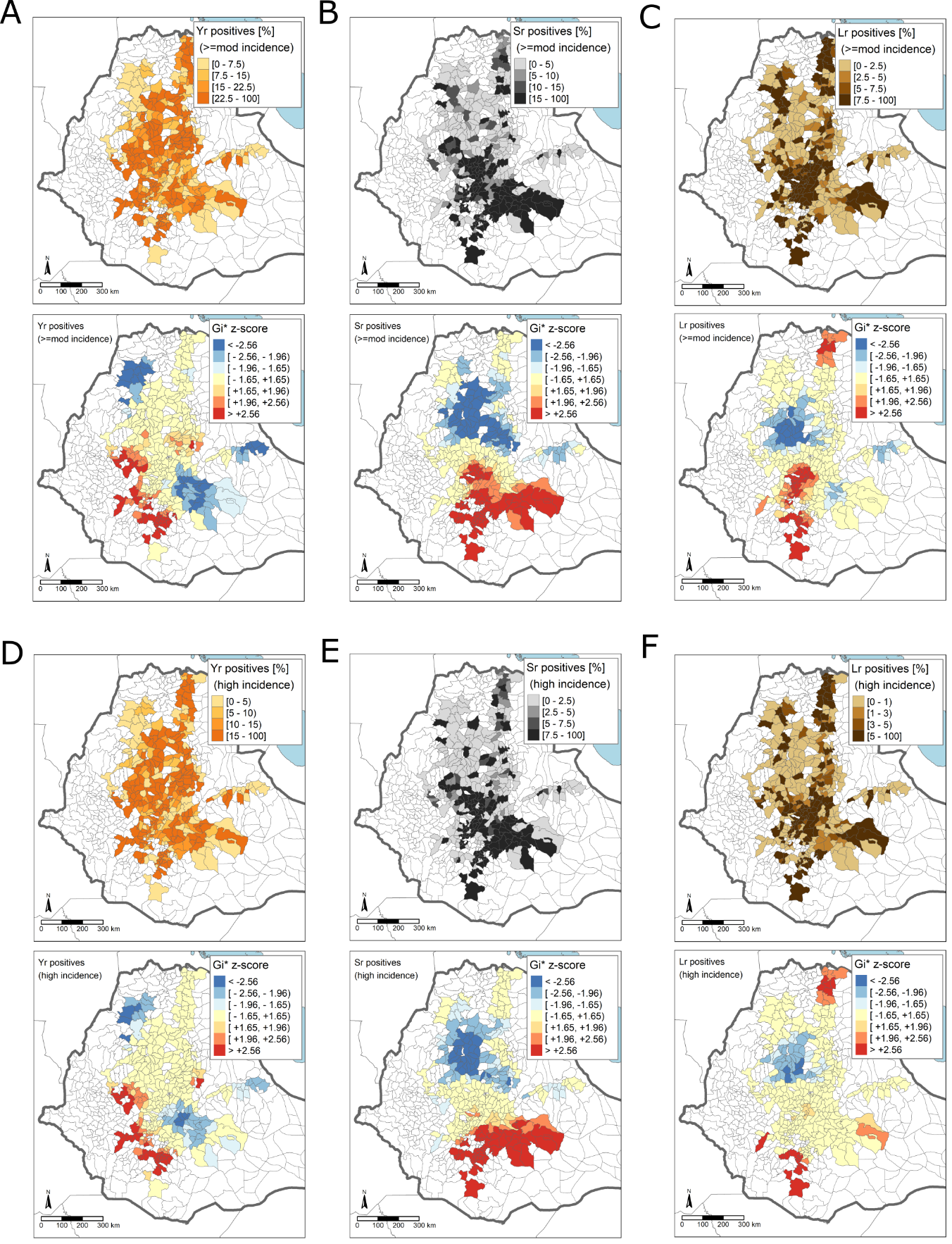
[S1 Fig. Spatial patterns of wheat rust outbreaks in Ethiopia in years 2010-2019 (moderate and high incidence cases). (A)** stripe rust, **(B)** wheat stem rust, **(C)** wheat leaf rust. **(A-C: top row):** proportion of moderate rust incidence cases (number of surveys with moderate or high incidence scores / total number of surveys) per district; **(A-C: bottom row)** hot- and cold-spots with respect to the proportion of moderate or high rust incidence cases per district. **(D)** stripe rust, **(E)** wheat stem rust, **(F)** wheat leaf rust. **(D-F: top row):** proportion of high rust incidence cases (number of surveys with high incidence scores / total number of surveys) per district; **(D-F: bottom row)** hot- and cold-spots with respect to the proportion of high rust incidence cases per district. Maps created using R as GIS [18-22].
