# Supplementary material for: Wheat rust epidemics damage Ethiopian wheat production: A decade of field disease surveillance reveals national-scale trends in past outbreaks
Source: PLoS One. 2021 Feb 3;16(2):e0245697. doi: 10.1371/journal.pone.0245697 (PMC7857641; doi:10.1371/journal.pone.0245697)
Supplement: S3 Fig — (top row) Proportion of low (left map), moderate (centre map) and high (right map) severity cases per district (calculated as: [number of surveys with disease severity score x / total number of surveys per district]). (bottom row) hot- and cold-spots of districts with high proportions of low (left map), moderate (centre map) and high (right map) stem rust severity cases. Maps created using R as GIS [18–22]. (DOCX) [file pone.0245697.s003.docx]

**
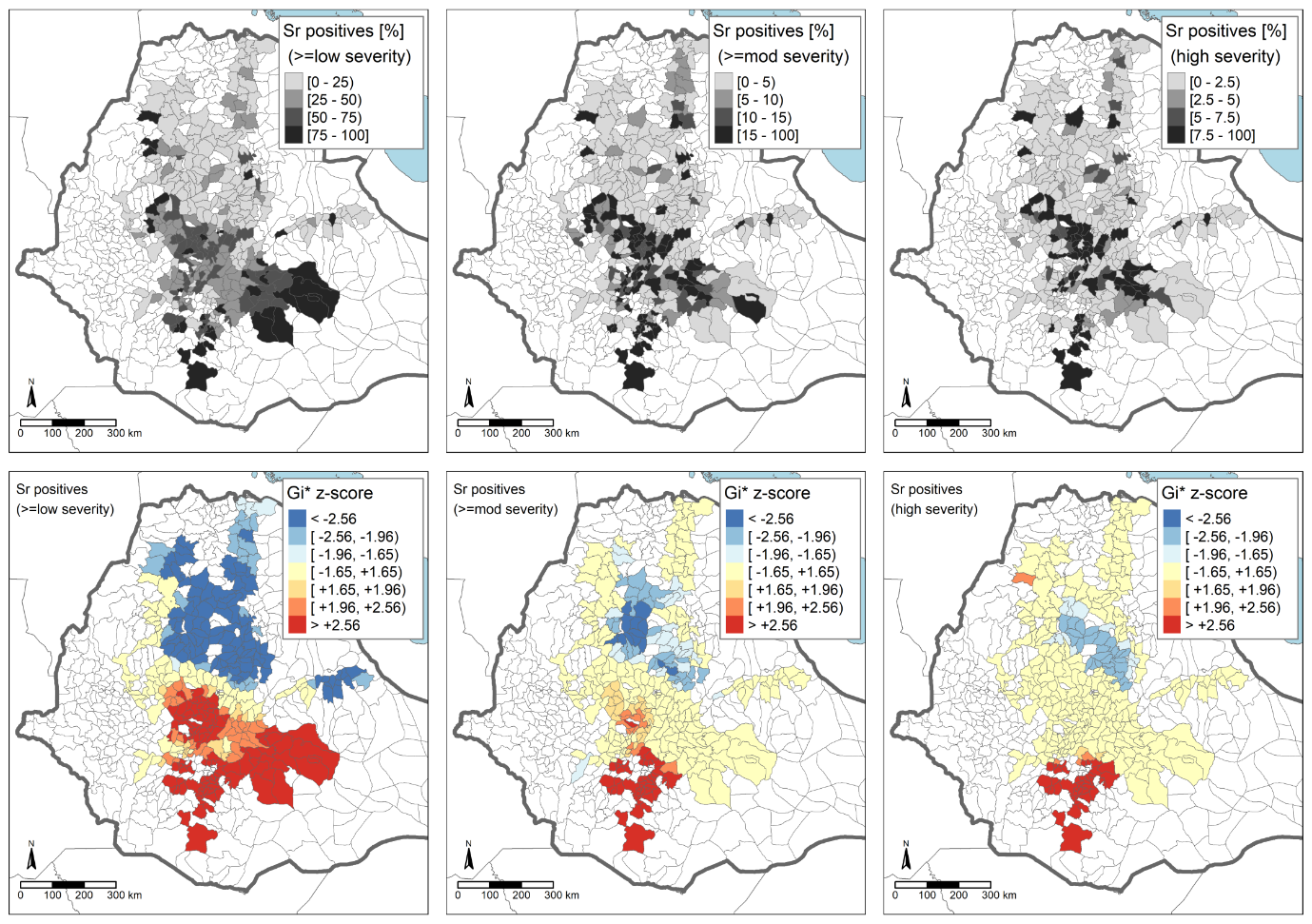
**

**S3 Fig. Spatial patterns of wheat stem rust outbreaks in Ethiopia in years 2010-2019 (severity scores). (top row)** Proportion of low (left map), moderate (centre map) and high (right map) severity cases per district (calculated as: [number of surveys with disease severity score x / total number of surveys per district]). **(bottom row)** hot- and cold-spots of districts with high proportions of low (left map), moderate (centre map) and high (right map) stem rust severity cases. Maps created using R as GIS [18-22].
