# Supplementary material for: Wheat rust epidemics damage Ethiopian wheat production: A decade of field disease surveillance reveals national-scale trends in past outbreaks
Source: PLoS One. 2021 Feb 3;16(2):e0245697. doi: 10.1371/journal.pone.0245697 (PMC7857641; doi:10.1371/journal.pone.0245697)
Supplement: S5 Fig — (A) wheat stripe rust; (B) wheat stem rust; (C) wheat leaf rust. A set of latitude-intervals are defined covering all latitudes at which wheat is grown in Ethiopia (x-axis). The surveys from all years are grouped according to their latitude coordinate and the aggregated mean prevalence is calculated for each latitude interval. (top row) long-term mean prevalence of incidence scores per latitude, (bottom row) long-term mean prevalence of severity scores per latitude. The labels at the top of the x-axes show the total number of surveys per latitude bin. Prevalence is calculated as: [number of surveys with incidence score x / total number of surveys per altitude-interval]). (DOCX) [file pone.0245697.s005.docx]

**
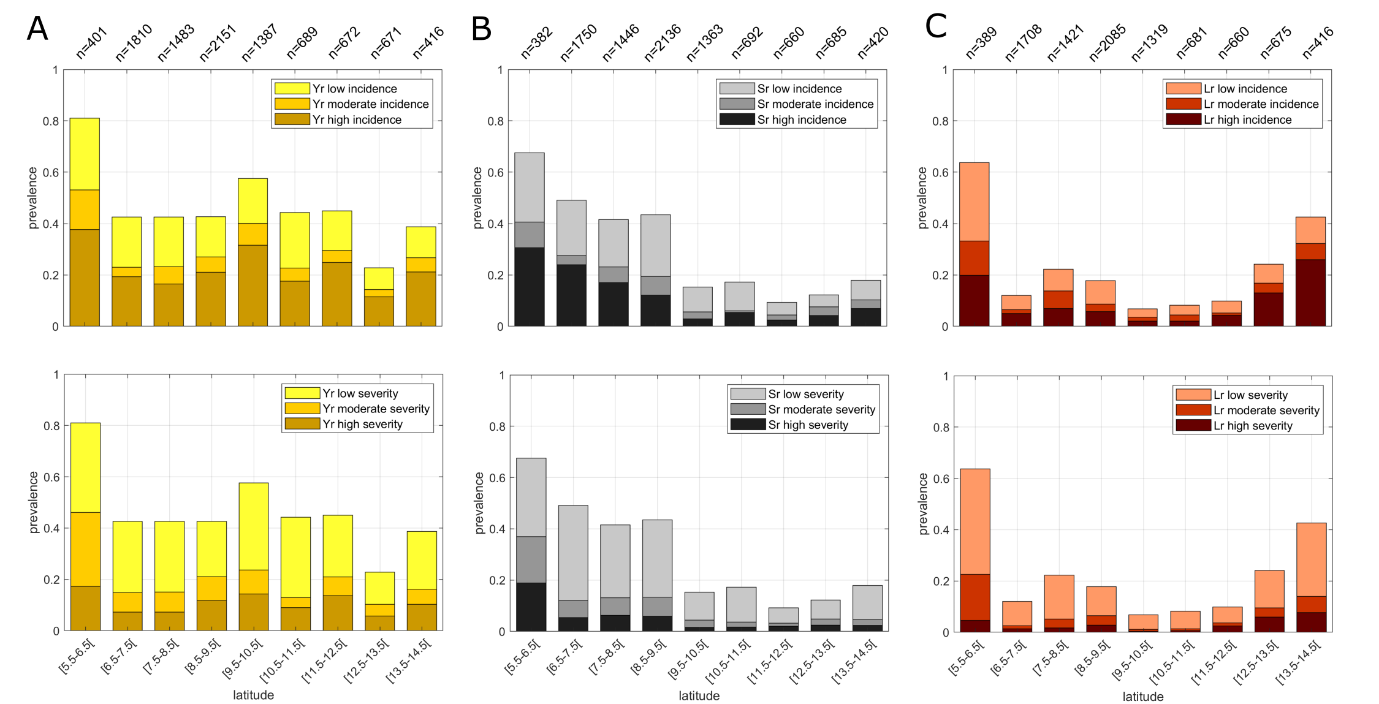
**

**S5 Fig. Latitudinal trends in wheat rust prevalence in Ethiopia in years 2010-2019. (A)** wheat stripe rust; **(B)** wheat stem rust; **(C)** wheat leaf rust. A set of latitude-intervals are defined covering all latitudes at which wheat is grown in Ethiopia (x-axis). The surveys from all years are grouped according to their latitude coordinate and the aggregated mean prevalence is calculated for each latitude interval. (top row) long-term mean prevalence of incidence scores per latitude, (bottom row) long-term mean prevalence of severity scores per latitude. The labels at the top of the x-axes show the total number of surveys per latitude bin. Prevalence is calculated as: [number of surveys with incidence score x / total number of surveys per altitude-interval]).
