# Supplementary material for: Wheat rust epidemics damage Ethiopian wheat production: A decade of field disease surveillance reveals national-scale trends in past outbreaks
Source: PLoS One. 2021 Feb 3;16(2):e0245697. doi: 10.1371/journal.pone.0245697 (PMC7857641; doi:10.1371/journal.pone.0245697)
Supplement: S7 Fig — (A) wheat stripe rust; (B) wheat stem rust; (C) wheat leaf rust. The labels at the top of the x-axes show the total number of surveys per year. Prevalence is calculated as: [number of surveys with disease incidence (severity) / total number of surveys per wheat variety]. (DOCX) [file pone.0245697.s007.docx]

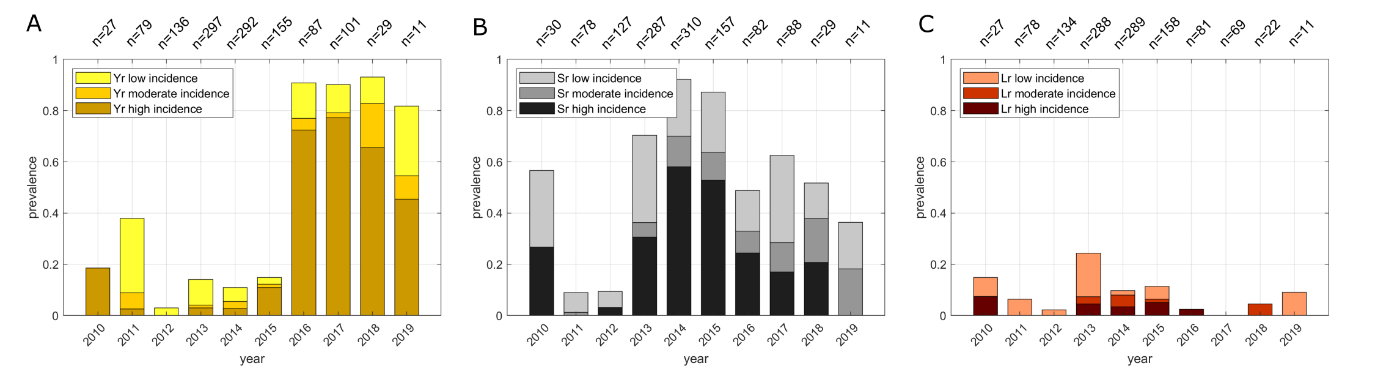


**S7 Fig. Interannual variations of disease prevalence (incidence scores) on wheat variety *Digalu*.** **(A)** wheat stripe rust; **(B)** wheat stem rust; **(C)** wheat leaf rust. The labels at the top of the x-axes show the total number of surveys per year. Prevalence is calculated as: [number of surveys with disease incidence (severity) / total number of surveys per wheat variety].
