# Supplementary material for: Wheat rust epidemics damage Ethiopian wheat production: A decade of field disease surveillance reveals national-scale trends in past outbreaks
Source: PLoS One. 2021 Feb 3;16(2):e0245697. doi: 10.1371/journal.pone.0245697 (PMC7857641; doi:10.1371/journal.pone.0245697)
Supplement: S8 Fig — (A) wheat stripe rust; (B) wheat stem rust; (C) wheat leaf rust. Symbols (circle, square, diamond) show sample mean prevalence levels in surveys and lines are linear fits to the mean prevalence levels (obtained using the MATLAB function fitlm). For each wheat rust and each disease metric (low incidence, moderate incidence, high incidence, low severity, moderate severity and high severity) it was tested if the slope of the fitted linear model is different from a constant line with a slope coefficient of zero using the MATLAB function CoefTest(). According to this test there is no statistically significant decrease or increase in the mean wheat stripe rust and wheat stem rust disease prevalence during years 2010–2019. The resulting p-values for the F-Test that the slope coefficient of the linear model is different to zero are larger than a significance level of 0.05 for all linear models of stripe rust and stem rust. However, there is a statistically significant decrease of wheat leaf rust prevalence over the years. The p-values for the F-Test of the slope of all leaf rust models are all smaller than 0.05 except for the linear fit to high severity levels. (DOCX) [file pone.0245697.s008.docx]

**
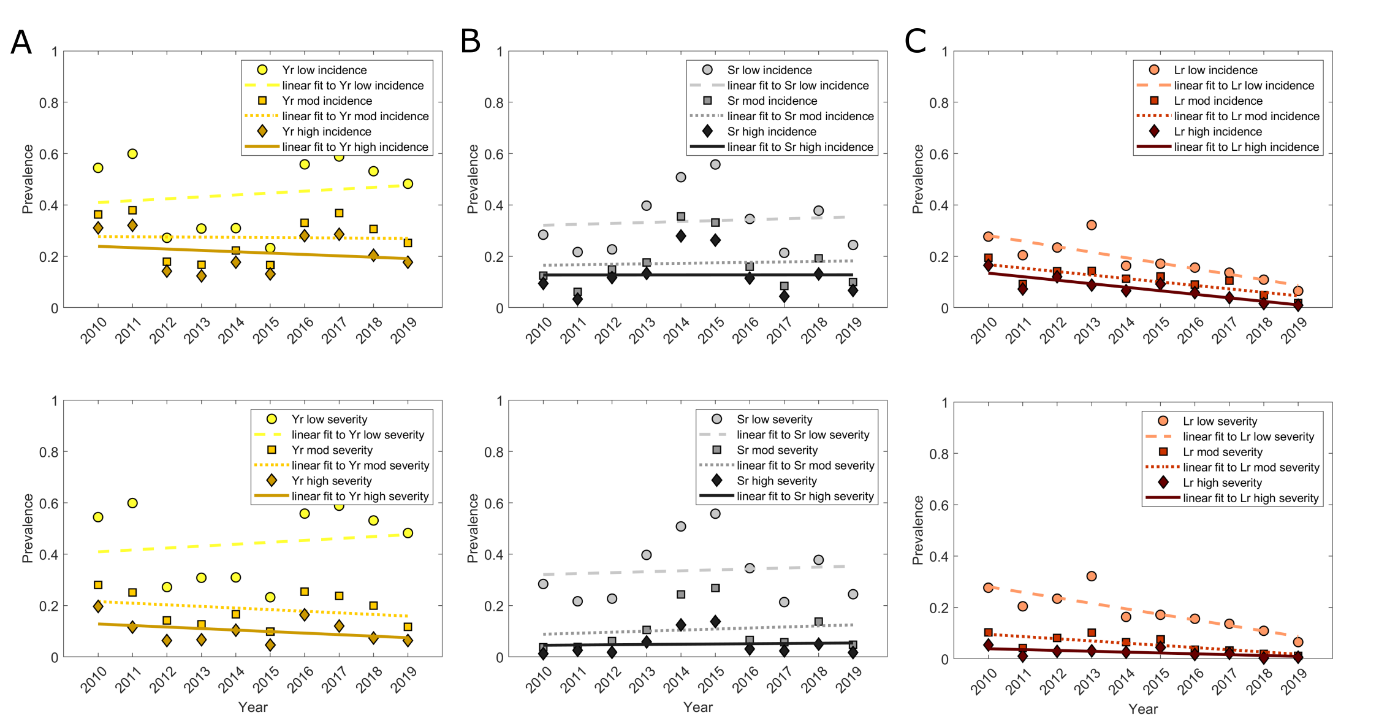
**

**S8 Fig. Assessing linear trends in interannual variations of wheat rust prevalence in Ethiopia in years 2010-2019. (A)** wheat stripe rust; **(B)** wheat stem rust; **(C)** wheat leaf rust. Symbols (circle, square, diamond) show sample mean prevalence levels in surveys and lines are linear fits to the mean prevalence levels (obtained using the MATLAB function *fitlm*). For each wheat rust and each disease metric (low incidence, moderate incidence, high incidence, low severity, moderate severity and high severity) it was tested if the slope of the fitted linear model is different from a constant line with a slope coefficient of zero using the MATLAB function *CoefTest*(). According to this test there is no statistically significant decrease or increase in the mean wheat stripe rust and wheat stem rust disease prevalence during years 2010 - 2019. The resulting p-values for the F-Test that the slope coefficient of the linear model is different to zero are larger than a significance level of 0.05 for all linear models of stripe rust and stem rust. However, there is a statistically significant decrease of wheat leaf rust prevalence over the years. The p-values for the F-Test of the slope of all leaf rust models are all smaller than 0.05 except for the linear fit to high severity levels.
