# Supplementary material for: Wheat rust epidemics damage Ethiopian wheat production: A decade of field disease surveillance reveals national-scale trends in past outbreaks
Source: PLoS One. 2021 Feb 3;16(2):e0245697. doi: 10.1371/journal.pone.0245697 (PMC7857641; doi:10.1371/journal.pone.0245697)
Supplement: S9 Fig — (A) wheat stripe rust; (B) wheat stem rust; (C) wheat leaf rust. The maps show disease incidence at all survey points of three exemplar years: 2010, 2014 and 2019. Symbols: green—no disease; yellow—low incidence; orange—moderate incidence; red—high incidence; grey areas—wheat producing regions. In 2010 a major wheat stripe rust epidemic led to infections covering large parts of western and central Ethiopian wheat producing areas (left map, A). In these regions, wheat stem rust incidence was low in 2010 (left map, B). In 2014 a major wheat stem rust epidemic occurred in large parts of southern and central Ethiopia (central map, B). In these areas wheat stripe rust incidence was low (central map, A). See S10 Fig for the corresponding severity scores at survey locations illustrated here. Maps created using R as GIS [18–22]. (DOCX) [file pone.0245697.s009.docx]

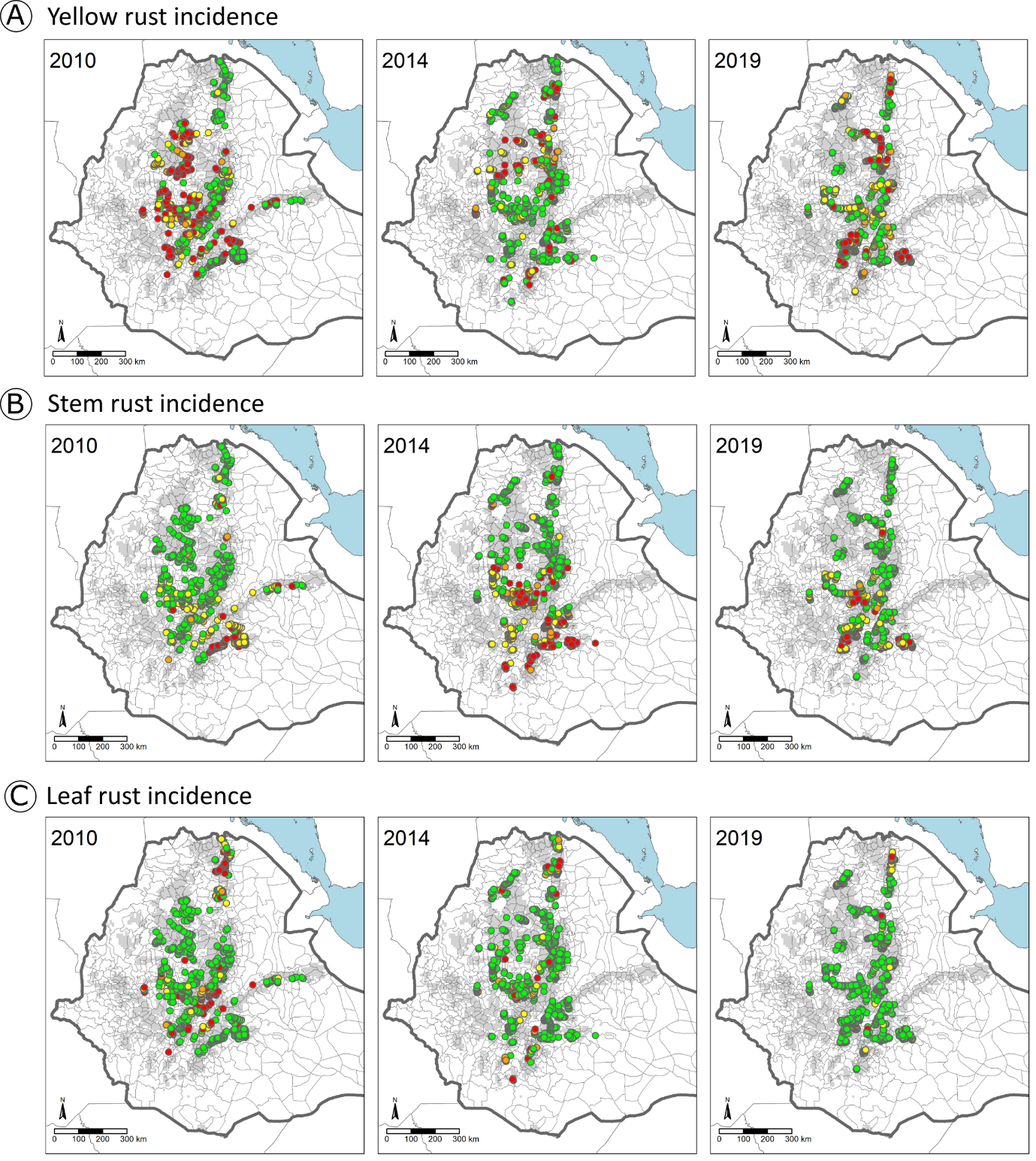


**S9 Fig. Interannual variations in the spatial patterns of wheat rust outbreaks in Ethiopia (incidence scores). (A)** wheat stripe rust; **(B)** wheat stem rust; **(C)** wheat leaf rust. The maps show disease incidence at all survey points of three exemplar years: 2010, 2014 and 2019. Symbols: green - no disease; yellow - low incidence; orange - moderate incidence; red - high incidence; grey areas - wheat producing regions. In 2010 a major wheat stripe rust epidemic led to infections covering large parts of western and central Ethiopian wheat producing areas (left map, A). In these regions, wheat stem rust incidence was low in 2010 (left map, B). In 2014 a major wheat stem rust epidemic occurred in large parts of southern and central Ethiopia (central map, B). In these areas wheat stripe rust incidence was low (central map, A). See S10 Fig for the corresponding severity scores at survey locations illustrated here. Maps created using R as GIS [18-22].
