# Supplementary material for: Wheat rust epidemics damage Ethiopian wheat production: A decade of field disease surveillance reveals national-scale trends in past outbreaks
Source: PLoS One. 2021 Feb 3;16(2):e0245697. doi: 10.1371/journal.pone.0245697 (PMC7857641; doi:10.1371/journal.pone.0245697)
Supplement: S11 Fig — The maps show disease incidence at all survey points at three different times of the main wheat season. Symbols: green—no disease; yellow—low incidence; orange—moderate incidence; red—high incidence; grey areas—wheat producing regions. (A) wheat stripe rust incidence at the beginning (left map), middle (centre map) and end (right map) of the main wheat season 2010; (B) wheat stem rust incidence at the beginning (left map), middle (centre map) and end (right map) of the main wheat season 2014; (C) wheat leaf rust incidence at the beginning (left map), middle (centre map) and end (right map) of the main wheat season 2010. See S12 Fig for the corresponding severity scores at survey locations illustrated here. Maps created using R as GIS [18–22]. (DOCX) [file pone.0245697.s011.docx]

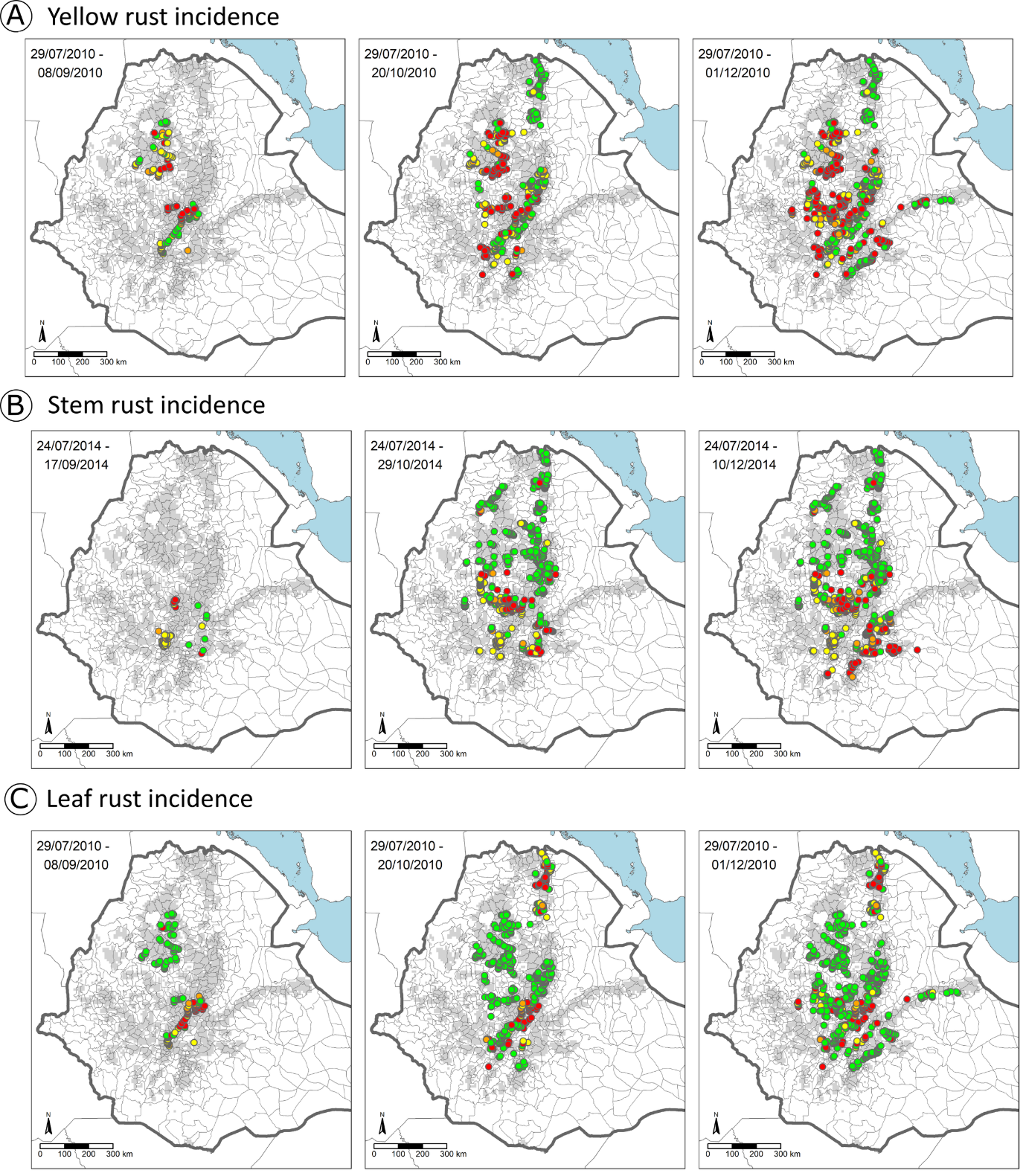


**S11 Fig. Within-season variations in spatial patterns of wheat rust outbreaks in Ethiopia (incidence scores).** The maps show disease incidence at all survey points at three different times of the main wheat season. Symbols: green - no disease; yellow - low incidence; orange - moderate incidence; red - high incidence; grey areas - wheat producing regions. **(A)** wheat stripe rust incidence at the beginning (left map), middle (centre map) and end (right map) of the main wheat season 2010; **(B)** wheat stem rust incidence at the beginning (left map), middle (centre map) and end (right map) of the main wheat season 2014; **(C)** wheat leaf rust incidence at the beginning (left map), middle (centre map) and end (right map) of the main wheat season 2010. See S12 Fig for the corresponding severity scores at survey locations illustrated here. Maps created using R as GIS [18-22].
