# Supplementary material for: Wheat rust epidemics damage Ethiopian wheat production: A decade of field disease surveillance reveals national-scale trends in past outbreaks
Source: PLoS One. 2021 Feb 3;16(2):e0245697. doi: 10.1371/journal.pone.0245697 (PMC7857641; doi:10.1371/journal.pone.0245697)
Supplement: S12 Fig — The maps show disease severity at all survey points at three different times of the main wheat season. Symbols: green—no disease; yellow—low incidence; orange—moderate incidence; red—high incidence; grey areas—wheat producing regions. (A) wheat stripe rust severity at the beginning (left map), middle (centre map) and end (right map) of the main wheat season 2010; (B) wheat stem rust severity at the beginning (left map), middle (centre map) and end (right map) of the main wheat season 2014; (C) wheat leaf rust severity at the beginning (left map), middle (centre map) and end (right map) of the main wheat season 2010. Maps created using R as GIS [18–22]. (DOCX) [file pone.0245697.s012.docx]

*
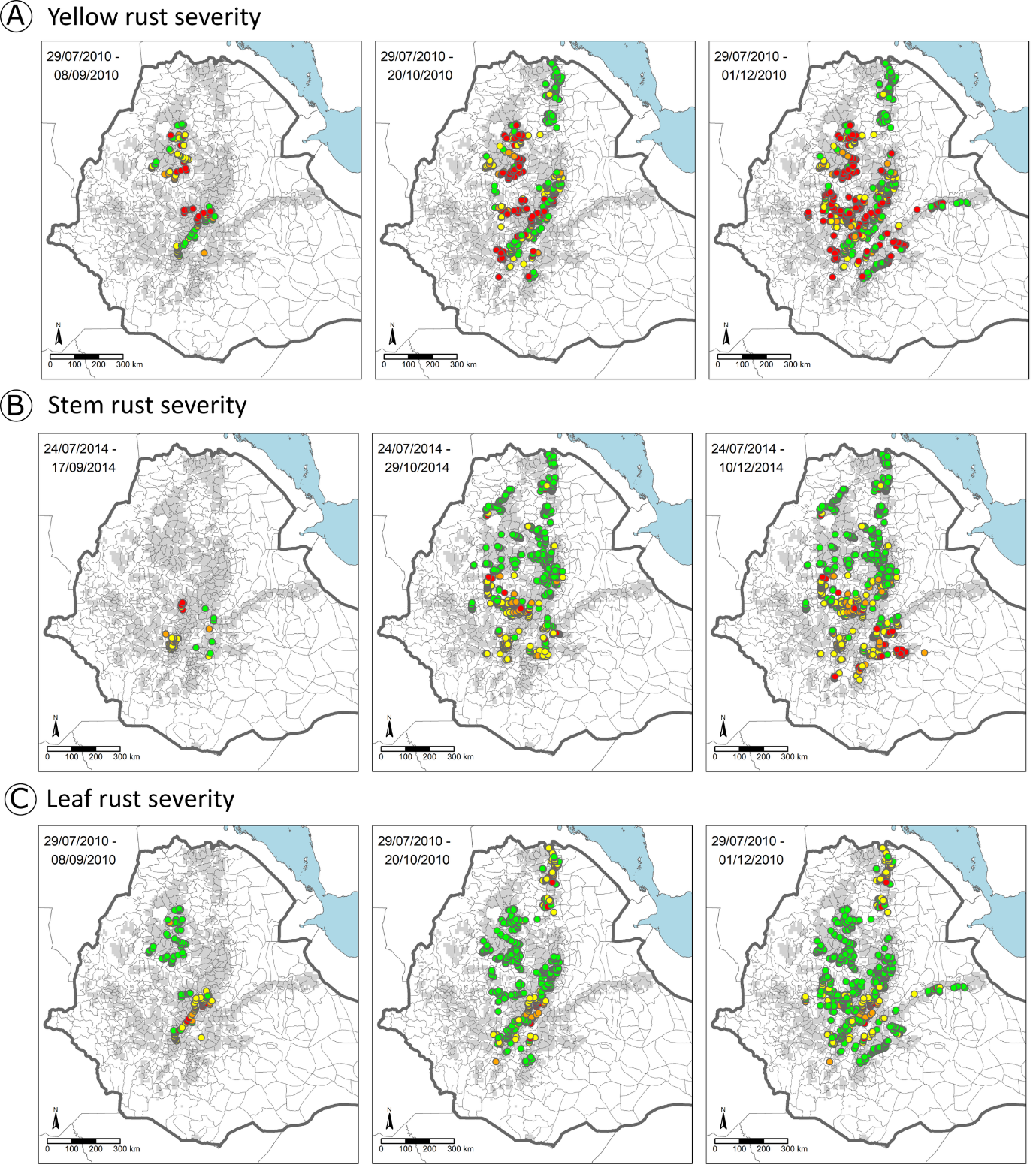
*

**S12 Fig. Within-season variations in spatial patterns of wheat rust outbreaks in Ethiopia (severity scores).** The maps show disease severity at all survey points at three different times of the main wheat season. Symbols: green - no disease; yellow - low incidence; orange - moderate incidence; red - high incidence; grey areas - wheat producing regions. **(A)** wheat stripe rust severity at the beginning (left map), middle (centre map) and end (right map) of the main wheat season 2010; **(B)** wheat stem rust severity at the beginning (left map), middle (centre map) and end (right map) of the main wheat season 2014; **(C)** wheat leaf rust severity at the beginning (left map), middle (centre map) and end (right map) of the main wheat season 2010. Maps created using R as GIS [18-22].
