# Supplementary material for: Wheat rust epidemics damage Ethiopian wheat production: A decade of field disease surveillance reveals national-scale trends in past outbreaks
Source: PLoS One. 2021 Feb 3;16(2):e0245697. doi: 10.1371/journal.pone.0245697 (PMC7857641; doi:10.1371/journal.pone.0245697)
Supplement: S13 Fig — (A) wheat stripe rust; (B) wheat stem rust; (C) wheat leaf rust. Two simple logistic models were used to predict moderate or high wheat rust incidence cases: A temporal model (Model 1, see Eq 2 in the main text) predicting wheat rust occurrence as a function of the time since the start of the main wheat season and a spatiotemporal model (Model 2, see Eq 3 in the main text), predicting wheat rust occurrence as a function of the time since the start of the main season and the location in Ethiopia (latitude, longitude and altitude). Model performance was tested by fitting the models to training data from all but 1 year of surveys and then conducting a ROC analysis for testing the performance of the fitted model against the data from the year not used for fitting (repeated for every year). The upper row shows the resulting AUC score of both models for each year and all rusts. The bottom row shows the corresponding ROC curves of one exemplar year. For example, the ROC curve of wheat stem rust (centre graph, bottom row) for the test-year 2013 corresponds to the AUC value of the model for wheat stem rust in year 2013 illustrated in the graph at the centre of the top row. For the analysis, all survey entries with moderate or high incidence values as “diseased” and all surveys with zero or low incidence as “healthy”. (DOCX) [file pone.0245697.s013.docx]

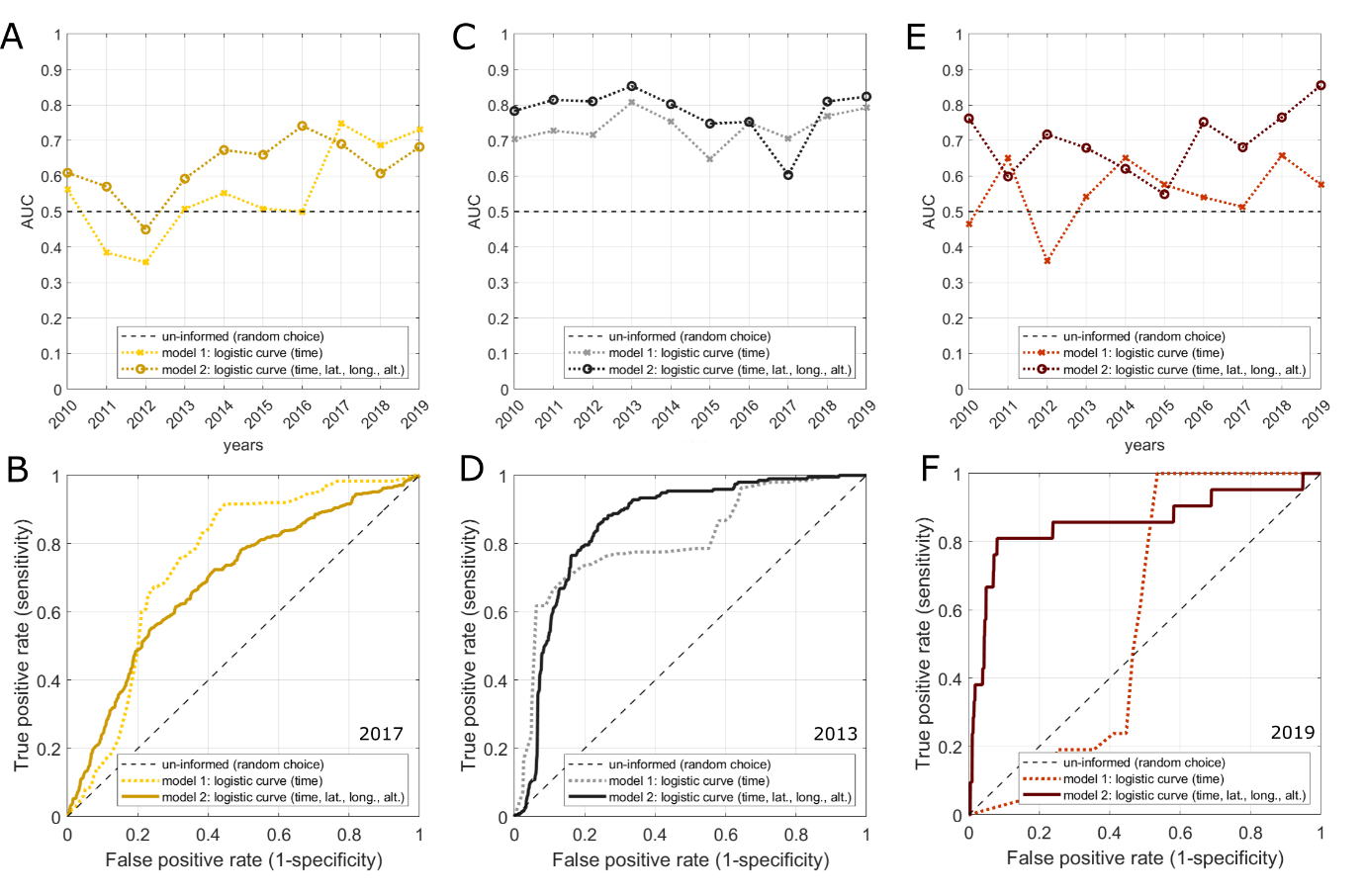


**S13 Fig. Performance of simple logistic models for predicting wheat rust occurrence in Ethiopia (moderate or high incidence cases). (A)** wheat stripe rust; **(B)** wheat stem rust; **(C)** wheat leaf rust. Two simple logistic models were used to predict moderate or high wheat rust incidence cases: A temporal model (Model 1, see Eq. 2 in the main text) predicting wheat rust occurrence as a function of the time since the start of the main wheat season and a spatiotemporal model (Model 2, see Eq. 3 in the main text), predicting wheat rust occurrence as a function of the time since the start of the main season and the location in Ethiopia (latitude, longitude and altitude). Model performance was tested by fitting the models to training data from all but 1 year of surveys and then conducting a ROC analysis for testing the performance of the fitted model against the data from the year not used for fitting (repeated for every year). The upper row shows the resulting AUC score of both models for each year and all rusts. The bottom row shows the corresponding ROC curves of one exemplar year. For example, the ROC curve of wheat stem rust (centre graph, bottom row) for the test-year 2013 corresponds to the AUC value of the model for wheat stem rust in year 2013 illustrated in the graph at the centre of the top row. For the analysis, all survey entries with moderate or high incidence values as “diseased” and all surveys with zero or low incidence as “healthy”.
