# Supplementary material for: Wheat rust epidemics damage Ethiopian wheat production: A decade of field disease surveillance reveals national-scale trends in past outbreaks
Source: PLoS One. 2021 Feb 3;16(2):e0245697. doi: 10.1371/journal.pone.0245697 (PMC7857641; doi:10.1371/journal.pone.0245697)
Supplement: S1 Table — (DOCX) [file pone.0245697.s014.docx]

|  | Model parameters | | | | | |  |
| --- | --- | --- | --- | --- | --- | --- | --- |
|  | $\alpha$ | | $\beta$ | | $\tau$ | | RMSE |
|  | estimate | SE | estimate | SE | estimate | SE |  |
| Wheat stripe rust |  |  |  |  |  |  |  |
| low incidence | 0.48 | 0.14 | 0.27 | 0.95 | -4.44 | 16.75 | 0.09 |
| moderate incidence | 0.31 | 0.06 | 0.64 | 0.69 | 1.75 | 1.29 | 0.09 |
| high incidence | 0.25 | 0.05 | 0.69 | 0.68 | 2.14 | 1.19 | 0.08 |
| low severity | 0.48 | 0.14 | 0.27 | 0.95 | -4.44 | 16.75 | 0.09 |
| moderate severity | 0.23 | 0.049 | 0.67 | 0.70 | 2.35 | 1.36 | 0.09 |
| high severity | 0.11 | 0.03 | 0.50 | 1.13 | 0.54 | 3.26 | 0.05 |
|  |  |  |  |  |  |  |  |
| Wheat stem rust |  |  |  |  |  |  |  |
| low incidence | 0.72 | 0.14 | 0.50 | 0.18 | 5.34 | 1.07 | 0.08 |
| moderate incidence | 0.44 | 0.09 | 0.64 | 0.26 | 5.97 | 0.95 | 0.06 |
| high incidence | 0.39 | 0.08 | 0.65 | 0.24 | 6.58 | 0.94 | 0.04 |
| low severity | 0.72 | 0.14 | 0.50 | 0.18 | 5.34 | 1.07 | 0.08 |
| moderate severity | 0.25 | 0.08 | 0.91 | 0.85 | 5.62 | 1.32 | 0.08 |
| high severity | 0.11 | 0.04 | 1.06 | 1.44 | 5.45 | 1.55 | 0.05 |
|  |  |  |  |  |  |  |  |
| Wheat leaf rust |  |  |  |  |  |  |  |
| low incidence | 0.21 | 0.04 | 2.48 | 4.89 | 1.83 | 0.82 | 0.11 |
| moderate incidence | 0.12 | 0.02 | 3.55 | 7.01 | 1.76 | 0.63 | 0.05 |
| high incidence | 0.07 | 0.01 | 3.58 | 4.39 | 1.56 | 0.60 | 0.03 |
| low severity | 0.21 | 0.04 | 2.48 | 4.89 | 1.82 | 0.82 | 0.11 |
| moderate severity | 0.08 | 0.02 | 0.65 | 0.88 | 2.54 | 1.84 | 0.03 |
| high severity | 0.02 | 0.003 | 4.18 | 5.09 | 1.58 | 0.57 | 0.01 |

**S1 Table: Parameter estimates for the univariate logistic model for within-season disease progress of wheat rusts in Ethiopia.** Eq. 2 (main text) is fitted to the survey data using nonlinear regression, as implemented in the Matlab function *fitnml*. The model is fitted separately for each rust and disease score (low, moderate and high incidence/severity). SE denotes the standard error of the parameter estimates. RMSE is the root mean squared error of the model fit.
