# Supplementary material for: Wheat rust epidemics damage Ethiopian wheat production: A decade of field disease surveillance reveals national-scale trends in past outbreaks
Source: PLoS One. 2021 Feb 3;16(2):e0245697. doi: 10.1371/journal.pone.0245697 (PMC7857641; doi:10.1371/journal.pone.0245697)
Supplement: S2 Table — (DOCX) [file pone.0245697.s015.docx]

|  | Model 1 | | | | Model 2 | | | |
| --- | --- | --- | --- | --- | --- | --- | --- | --- |
|  | data: low  positives | | data: moderate positives | | data: low  positives | | data: moderate positives | |
|  | incidence | severity | incidence | severity | incidence | severity | incidence | severity |
| stripe rust |  |  |  |  |  |  |  |  |
| mean AUC | 0.52 | 0.52 | 0.57 | 0.58 | 0.60 | 0.60 | 0.63 | 0.63 |
| mean accuracy | 0.49 | 0.49 | 0.56 | 0.57 | 0.62 | 0.62 | 0.63 | 0.61 |
| stem rust |  |  |  |  |  |  |  |  |
| mean AUC | 0.71 | 0.71 | 0.74 | 0.74 | 0.78 | 0.78 | 0.78 | 0.77 |
| mean accuracy | 0.71 | 0.71 | 0.69 | 0.69 | 0.71 | 0.71 | 0.74 | 0.75 |
| leaf rust |  |  |  |  |  |  |  |  |
| mean AUC | 0.54 | 0.54 | 0.55 | 0.60 | 0.66 | 0.66 | 0.70 | 0.70 |
| mean accuracy | 0.52 | 0.52 | 0.48 | 0.49 | 0.61 | 0.61 | 0.70 | 0.68 |

**S2 Table: Performance of simple empirical models for predicting wheat rust occurrence in Ethiopia.** Model 1 refers to Eq. 2 and Model 2 refers to Eq. 3 in the main text. For the testing summarized in the column “data: low positives” all survey data with disease scores >= low incidence/severity was categorized as “diseased”. For the testing summarized in the column “data: moderate positives” all survey data with disease scores >= moderate incidence/severity was categorized as “diseased”. The mean performance metrics, AUC value and accuracy, are calculated from 10 repetitions of model testing with varying subsets of training and test data.
