# Supplementary material for: Wheat rust epidemics damage Ethiopian wheat production: A decade of field disease surveillance reveals national-scale trends in past outbreaks
Source: PLoS One. 2021 Feb 3;16(2):e0245697. doi: 10.1371/journal.pone.0245697 (PMC7857641; doi:10.1371/journal.pone.0245697)
Supplement: S1 Appendix — (DOCX) [file pone.0245697.s028.docx]

**S1 Appendix: Spatial analysis of wheat rust outbreaks in Ethiopia**

**S1.1** Test spatial autocorrelation of wheat rust prevalence in administrative districts of Ethiopia

The global *Morans-I* statistic was calculated using the R function *moran.test()* from the package *spdep*. As input data, the aggregated disease scores per administrative district are used (Fig 1G-I, main text; S1-S4 Figs). For each district, only direct neighbours are considered using a binary weighting. The statistic was calculated separately for each rust and each disease level (>=low, >=moderate, high incidence and severity).

Results indicate that the null hypothesis of random spatial distribution of disease (with respect to the proportion of positives) per administrative district can be rejected with p-values <<0.01 for all rusts and all disease levels (>= low incidence, >= moderate incidence, high incidence; >= low severity, >= moderate severity and high severity), with two exceptions: there is no significant spatial autocorrelation with respect to the proportion of high stripe and leaf rust cases per administrative district. Districts with high proportions of high incidence/severity stripe and leaf rust cases appear randomly distributed in space. For some districts, the total number of available surveys and particularly the total number of high disease incidence/severity cases is low, which means district-level proportions of disease are only crude estimates of actual disease levels.

**S1.2.** Analysis of hot- and cold-spots of wheat rust prevalence in Ethiopia

For the identification of hot- and cold-spots the *Getis-Ord Gi** statistic is calculated using the function *localG()* of the R package *spdep*. As input data, the proportion of positives per administrative district is used (Fig 1G-I, main text; S1-S4 Figs). All neighbouring districts within a geographic distance of 1 decimal degree (approx. 100 kilometres) are considered. The statistic was calculated separately for each rust and disease level (low, moderate, high incidence and severity).

Results indicate clusters of districts (hot-spots, cold-spots) with higher (or lower) proportion of positives than expected under the assumption of random spatial distribution. Hot- and cold-spots were calculated separately for each wheat rust and all categories of incidence and severity (see S1-S4 Figs). Also, hot- and cold-spots were analysed using different cut-off distances for defining the “neighbourhood” of districts. As can be expected, the geographic extent of “hot- and cold-spots” is sensitive to the distance chosen to define “neighbouring” districts. Within a range of approximately 50 to 250 km, hot- and cold-spots are broadly comparable to those shown in Fig 1 in the main text. This indicates the following spatial characteristics in past outbreak patterns are robust: there are pronounced differences in the mean spatial distribution of wheat rusts in different geographic areas of Ethiopia; wheat stem rust prevalence is substantially higher in southern districts; stripe rust prevalence is higher in central-western and south-western districts and stripe rust prevalence is higher in a few northern districts as well as in the south-west. Inferring more detailed spatial trends is challenging given the limited amount of survey data.

For some districts, the number of surveys is very low (e.g. southern-most districts), hence the proportion of positives per district are only crude estimates and may not be representative of actual disease prevalence in these districts, which could lead to a misleading visual bias in patterns of hot- and cold-spots. However, the key longitudinal and latitudinal trends were additionally confirmed by calculating mean prevalence levels at a set of latitudes and longitudes (see S5 and S6 Figs).
