# Supplementary material for: Wheat rust epidemics damage Ethiopian wheat production: A decade of field disease surveillance reveals national-scale trends in past outbreaks
Source: PLoS One. 2021 Feb 3;16(2):e0245697. doi: 10.1371/journal.pone.0245697 (PMC7857641; doi:10.1371/journal.pone.0245697)
Supplement: S2 Appendix — (DOCX) [file pone.0245697.s029.docx]

**S2 Appendix: Estimating approximate financial losses caused by wheat rusts in Ethiopia**

The following paragraphs describe how we estimate financial losses caused by wheat rusts in Ethiopia (as a supplement to Eq. 1 and the text in the main manuscript).

**S2.1.** Approximation of the total wheat area infected with wheat rusts in Ethiopia

The total area of wheat moderately or highly infected with wheat rust, *r*, in year, *t*, is estimated as

Eq. S1: $A_{rt}\left( i \right){= \overline{A}}_{t}\times p_{rt} (i)$

where: $\overline{A}_{t}$ [ha] denotes the total area of wheat harvested in year, *t*, in Ethiopia, as given in FAO statistics [1] and $p_{rt} (i)$ denotes the proportion of the total wheat area infected with wheat rust, *r*, in year, *t*. The proportion of the total wheat area infected with wheat rusts is estimated based on incidence scores, *i*, in surveys as

Eq. S2: $p_{rt}(i)=$ ( $f_{rt}^{i=high}$ $\times s_{rt}^{i=high})+( f_{rt}^{i=mod}$ $\times s_{rt}^{i=mod}$)

where: $f_{rt}^{i}$ denotes the annual proportion of fields with moderate (*i* = mod) and high (*i* = high) infections relative to all surveyed fields in year, *t*, for rust type, *r* (calculated as: [number of fields with infection / total number of fields surveyed]); and $s_{rt}^{i}$ denotes the annual mean proportion of the area of infected fields covered with wheat rusts (the incidence categories reported in surveys for each field provide percentage estimates of the field area that is infected with rusts). The infected area is approximated separately for each type of wheat rust, r.

**S2.2.** Approximation of yield loss caused by wheat rusts on infected areas

The yield loss caused by wheat rust infections is calculated as

Eq. S3: $Y_{rt}(s, g)=l_{rt}(s,g) \times\overline{Y}_{t}$,

where, $\overline{Y}_{t}$, denotes the average wheat yield in year, t, in Ethiopia, as given in FAO statistics, and, $l_{rt}$, denotes the approximate percentage of yield lost due to wheat rust ($r$) infections on infected areas. The percentage of yield loss caused by rusts is calculated separately for each type of wheat rust, *r*, based on the survey data about disease severity, *s*, and wheat growth stage, *g*, combined with empirical relations from past experiments [25]. For each surveyed wheat field in year, t, we approximate yield losses caused by wheat rusts as follows:

1. read the survey data about wheat rust severity and wheat growth stage (only considering moderate or high severity cases). When no information about wheat growth stage is available, we use the average growth stage of all surveys of year, *t*.
2. approximate the yield loss on each surveyed field as a function of wheat rust disease severity and growth stage by using the following published empirical relations:
   1. for wheat stem rust: a previous empirical study has analysed yield loss as a function of wheat growth stage and wheat rust severity ([25], table 23, p. 51). We use these empirical results and obtain yield loss values for the combination of growth stage and disease severity reported in field surveys by looking up the corresponding values in table 23 in [25] (linear interpolation for values between the categories provided in table 23) - assuming that yield loss in Ethiopian wheat fields may be approximated by the yield loss measured in these aforementioned experiments.
   2. for leaf rust: similar approximation method as described above for wheat stem rust but using the empirical values from past experiments measuring yield losses caused by wheat leaf rust, as reported in table 24 (in [25], p. 51).
   3. for wheat stripe rust: as for wheat stem rust and wheat leaf rust, [25] also describes an empirical relation between yield loss, wheat growth stage and disease severity for wheat stripe rust ([25], p. 59). This is used to calculate yield loss as a function of wheat growth stage and disease severity from field surveys.
3. we obtain the annual mean fraction of yield loss by averaging over the loss fraction in all individual fields.
